# Enhancing the Efficacy of Drug-loaded Nanocarriers against Brain Tumors by Targeted Radiation Therapy

**DOI:** 10.18632/oncotarget.777

**Published:** 2013-12-23

**Authors:** Brian C. Baumann, Gary D. Kao, Abdullah Mahmud, Takamasa Harada, Joe Swift, Christina Chapman, Xiangsheng Xu, Dennis E. Discher, Jay F. Dorsey

**Affiliations:** ^1^ Department of Radiation Oncology, Perelman School of Medicine at the University of Pennsylvania, Philadelphia, PA; ^2^ NanoBio-Polymers Laboratory, Department of Chemical and Biomolecular Engineering, University of Pennsylvania, Philadelphia, PA

**Keywords:** glioblastoma multiforme, nanocarrier, radiation therapy, brain tumors, chemotherapy

## Abstract

Glioblastoma multiforme (GBM) is a common, usually lethal disease with a median survival of only ~15 months. It has proven resistant in clinical trials to chemotherapeutic agents such as paclitaxel that are highly effective in vitro, presumably because of impaired drug delivery across the tumor's blood-brain barrier (BBB). In an effort to increase paclitaxel delivery across the tumor BBB, we linked the drug to a novel filomicelle nanocarrier made with biodegradable poly(ethylene-glycol)-block-poly(ε-caprolactone-r-D,L-lactide) and used precisely collimated radiation therapy (RT) to disrupt the tumor BBB's permeability in an orthotopic mouse model of GBM. Using a non-invasive bioluminescent imaging technique to assess tumor burden and response to therapy in our model, we demonstrated that the drug-loaded nanocarrier (DLN) alone was ineffective against stereotactically implanted intracranial tumors yet was highly effective against GBM cells in culture and in tumors implanted into the flanks of mice. When targeted cranial RT was used to modulate the tumor BBB, the paclitaxel-loaded nanocarriers became effective against the intracranial tumors. Focused cranial RT improved DLN delivery into the intracranial tumors, significantly improving therapeutic outcomes. Tumor growth was delayed or halted, and survival was extended by &gt;50% (p<0.05) compared to the results obtained with either RT or the DLN alone. Combinations of RT and chemotherapeutic agents linked to nanocarriers would appear to be an area for future investigations that could enhance outcomes in the treatment of human GBM.

## INTRODUCTION

Over 20,000 Americans are diagnosed annually with glioblastoma multiforme (GBM) [1]. Standard treatment usually consists of surgery, radiation therapy (RT), and temozolomide chemotherapy, but median survival is only about 14.6 months [2]. Given these unsatisfactory results, new therapeutic approaches are clearly needed. Although other chemotherapeutic drugs such as paclitaxel (Taxol) are even more effective against GBM cells *in vitro* than temozolomide [3], they have been found to be clinically ineffective against GBM [4]. The limited efficacy of such drugs has been attributed to an inability to achieve therapeutic concentrations of these drugs in the tumor due to the presence of the blood-brain barrier (BBB) – specifically the BBB within the tumor. Modulation of both drug delivery and the integrity of the BBB thus represent promising strategies for enhancing treatment efficacy.

Solid tumors often have vascular systems that are leaky and have impaired blood flow compared to the circulation through normal tissue. Structural characteristics of tumor vascularity such as increased tortuosity, irregular shape and dilation of blood vessels coupled with endothelial fenestrations result in leakage of blood plasma macromolecules and drugs into tumor tissue. The extravasation of these plasma macromolecules into tumors and their concentration and retention within the tumor is a phenomenon referred to as the enhanced permeability and retention (EPR) effect [5, 6]. The EPR effect is the basis, for example, of preferential uptake of gadolinium contrast agents into tumors compared to normal brain tissue, as observed by magnetic resonance imaging (MRI). The anatomical and physiological factors promoting the EPR effect that lead to increased extravasation of drugs and macromolecules from the serum into tumors are not uniformly distributed throughout tumors [7, 8]. The EPR effect is often maximal at core regions within a tumor, regions frequently characterized by necrosis, while the EPR effect is diminished at the peripheral zones of a tumor. These peripheral areas may contain many viable cancer cells and are also the regions where the tumor BBB is most likely to remain intact.

A novel approach to maximize EPR-driven concentration of chemotherapeutic agents within tumors is utilization of drug-loaded nanocarriers (DLNs) that stably incorporate drug molecules [9, 10]. These agents offer the potential to increase drug delivery into tumors by either reducing drug clearance/excretion to increase the drug's serum half-life or by enhancing permeability of the nanocarrier-drug combination through the tumor's abnormal endothelium compared to the permeability of the drug alone.

One class of recently developed DLN is filomicelles that are filamentous, polymeric self-assemblies that can incorporate paclitaxel. Filomicelles avoid rapid clearance by the mononuclear phagocytic system of the liver and spleen, causing an increase in the serum half-life of the drug [11, 12]. Flexibility of the filaments was shown to be important in reducing drug clearance, and the crystalline rigidity of past polymer assemblies is suppressed in these filomicelles with novel hydrophilic-*linked*-hydrophobic *block* copolymers of poly(ethylene oxide)-*block*-poly(ε-caprolactone-random-D,L-lactide) (OCLA) that randomize the packing in the hydrophobic, polyester core.In subcutaneous tumor xenografts, this reduction in drug clearance has been shown to maximize drug deliver to the tumors with enhanced anti-tumor efficacy [11].

Filomicelles have been shown to flow through the brain vasculature, but like other DLN they do not penetrate the BBB [13]. Therefore, targeted disruption of the tumor's BBB to increase DLN permeability might enhance the effectiveness of these and other DLNs for treating intracranial tumors. Conventional approaches for transiently disrupting the BBB before delivery of chemotherapy, such as efflux transporter inhibitors, bradykinins, intra-arterial infusions of osmotic disruption agents (e.g. mannitol), and convection-enhanced delivery mechanisms [14-19], lack specificity for tumor-associated vasculature. Non-targeted disruption of the BBB with these agents is likely to facilitate accumulation of drugs into the normal brain, resulting in potentially serious normal tissue complications limiting the dose that can be used for treatments [14-18]. Several approaches for more targeted disruption of the tumor-BBB are being studied in pre-clinical models with some success, including the use of focused ultrasound as well as vascular-active agents like TNF-alpha that appear to preferentially enhance tumor-BBB disruption to facilitate the delivery of chemotherapeutics [7, 10, 20]. Another such approach is targeted radiation therapy (RT) which offers an ideal solution for focally disrupting the tumor BBB while minimizing disruption of the adjacent normal brain's BBB as RT directed at intracranial targets modulates the BBB with specificity by causing endothelial cell dysfunction only within the radiation portal [21-27].

RT targeting of tumors has become increasingly precise in recent years with rapid advances in imaging, computer modeling, beam generation, and conformal dose delivery. The safety record of RT also spans many decades; most patients with solid malignancies now undergo RT at some point during their treatment, resulting in cure or therapeutic response in both primary and metastatic tumors. For intracranial tumors such as GBM, RT has become a mainstay of treatment. Surprisingly, strategies that exploit RT's impact on brain tumor BBB permeability in order to enhance the efficacy of anti-cancer drugs remain largely under-explored [22, 28].

To investigate the potential advantages of cranial RT to increase the potency of DLN in the treatment of brain tumors, we used a novel mouse-based model of human GBM to evaluate the relative efficacy of combined modality treatment with RT plus a nanocarrier filomicelle loaded with paclitaxel compared to single modality treatment with either RT, drug loaded nanocarrier, or the nanocarrier without paclitaxel. Paclitaxel is an ideal therapeutic agent for such a trial. It is insoluble in water, widely used against many cancers, and is even more efficacious against GBM *in vitro* than temozolomide [3], yet it is ineffective *in vivo* against intracranial tumors both in animal models and in clinical trials of patients who also received RT, presumably due to the inability of the drug in its free form to penetrate the tumor BBB [4, 29-32]. The poor response of brain tumors to paclitaxel alone *in vivo* ensures that any response to the paclitaxel-loaded nanopolymer could not be ascribed to dissociation of the paclitaxel from the nanocarrier or to degradation of the carrier.

**Figure 1:**
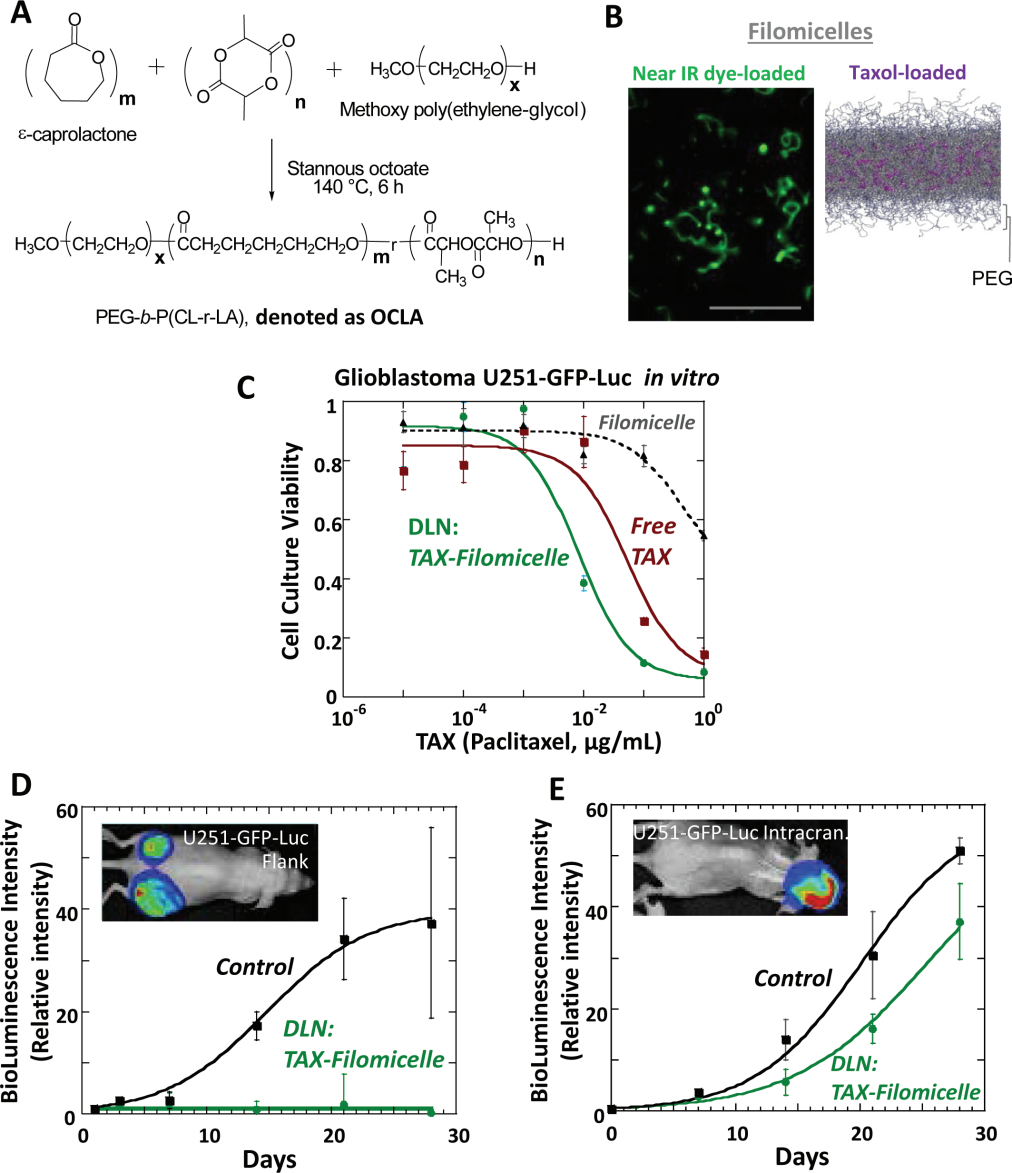
Drug-loaded nanocarriers consisting of polymeric filomicelles that incorporate paclitaxel (Taxol) prove effective against GBM in cell culture and in flank tumors but appear ineffective against intracranial tumors (A) Synthesis of block copolymer (OCLA) that assembles into filomicelle drug-loaded nanocarrier (DLN) employed in this study. (B) Near-infrared microscopy of dye-loaded filomicelles and anti-cancer drug paclitaxel loaded into filomicelles by molecular dynamics simulation. Bar indicates scale 20 μm. (C) Human-derived U251 cells stably expressing GFP and luciferase (“U251-GFP-Luc”) were exposed for 96 hours to the indicated concentrations of paclitaxel, the latter either in free form (“Free TAX”) or incorporated into nanofilomicelles (“TAX-Filomicelle”), or to the concentrations of the nanofilomicelles alone without paclitaxel (“Filomicelle”). The nanocarrier loaded with paclitaxel is at least as effective as free paclitaxel. (D-E) Paclitaxel-loaded nanocarriers (“DLN”) suppressed growth of flank tumors (D) far more effectively than intracranial tumors (E). Mice with either flank or intracranial tumors grown from U251-GFP-Luc cells were treated with paclitaxel-loaded DLN or the nanofilomicelles alone without paclitaxel (“Control”) and assessed by serial BLI. DLN-treated intracranial tumors showed minimal growth delay compared to Control mice treated with the nanofilomicelles lacking paclitaxel, consistent with the tumor-BBB as a barrier to delivery. Inset are images representing bioluminescent imaging of mice with either flank or intracranial tumors prior to treatment with DLN.

We had previously reported the efficacy of a paclitaxel-*linked* filomicelle nanocarrier for treating mice with subcutaneous tumor implants derived from a lung cancer cell line [11], but for experiments using human-derived GBM cells implanted into the brain we used a new, even more flexible, less crystalline OCLA filomicelle nanocarrier *linked* to paclitaxel (Fig. 1A, 1B, Suppl. Fig. 1, and Suppl. Text). Based on our previous work [11], we expected that this new nanocarrier would be more effective at avoiding clearance by the mononuclear phagocytic system than more rigid nanocarriers, thus prolonging the serum half-life of the drug which would increase its potential for diffusion through a tumor BBB disrupted by RT.

For our mouse model, we implanted human-derived U251 GBM cells. This cell line was selected because previous reports demonstrated that implants of these cells retain numerous characteristics frequently observed in human GBM tumors [33], including necrosis, regions of hypoxia, increased vascular endothelial growth factor (VEGF), HIF-1α expression and tumor invasiveness [33-37].

We evaluated the response of implanted tumors to the paclitaxel-loaded filomicelle both with and without RT using serial bioluminescent imaging (BLI) of GBM cells to track tumor growth and response to treatment. The treatment response was also serially assessed using clinically relevant measures such as body weight and overall survival. We quantified RT-modulation of the BBB via extravasation of Evans Blue dye, immunoglobulin (IgG), and DLN from the systemic circulation [14, 15, 38-40]. As controls, we also examined the effect of RT alone and the effect of the nanocarrier *linked* only to an inert, near-infrared (IR) dye that had demonstrated no anti-tumor activity in previous experiments [11].

## RESULTS

### Mouse model of human GBM suitable for serial imaging

Our studies used human GBM cells that retain numerous characteristics frequently observed in tumors from patients [33, 35-37]. In order to track the growth of these cells serially and to distinguish them from host tissues of the mouse, a lentivirus containing genes for luciferase and green fluorescent protein (GFP) was transduced into and stably expressed in U251 cells (Suppl. Fig. 2A). These “U251-GFP-Luc” cells were injected into the brains of mice via stereotactic techniques, and the resultant tumors and their growth were readily detectable in live mice via bioluminescent imaging (BLI) (Suppl. Fig. 2B) [47]. Sacrificed mice were assessed by fluorescent microscopy or hematoxylin and eosin staining (Suppl. Fig. 2C-E) that confirmed that the established tumors recapitulated many of the key features associated with glioblastoma, such as necrosis, regions of hypoxia, increased vascular endothelial growth factor (VEGF), and HIF-1α expression (Suppl. Fig 3) as previously reported by Radaelli et al [35]. We also saw evidence of tumor invasiveness (Suppl. Fig 7) with our mouse model consistent with the findings of others [35-37]. We further confirmed both *in vitro* and *in vivo* that the intensity of the bioluminescent signal was directly proportional to the number of viable luciferase-expressing GBM cells and to the physical dimensions of the tumor. Higher BLI signal correlated well with increased tumor cell burden (Suppl. Fig. 4-5 and data not shown) [34]. These studies established that our animal model of GBM recapitulated clinical features of human tumors yet was amenable to serial non-invasive imaging.

### Nanocarriers with paclitaxel are effective against GBM cells in culture and in flank xenografts but not against intracranial GBM tumors

Before assessing the efficacy of combined modality therapy with radiation plus DNL against GBM, we performed pilot studies to measure the efficacy of DLN alone against GBM cells in culture as well as against cells grown as xenografts in either the flank or the brain. We chose to incorporate paclitaxel into the DLN because this chemotherapy was effective against GBM cells in culture but is reportedly unable to penetrate the BBB.

Both free paclitaxel and paclitaxel-loaded filomicelles reduced the *in vitro* viability of U251, U251-GFP-Luc, and other GBM cells to a similar degree when normalized to an equivalent dose of free paclitaxel (Fig. 1C, Suppl. Table 1).

After cell culture studies confirmed the potential anti-GBM efficacy of our DLN, we tested its efficacy against U251-GFP-Luc tumors established either in the flank or orthotopic intracranial tumors. Tumors implanted at either site grew at identical rates and were amenable to serial bioluminescent imaging (Table 1, Suppl. Fig. 5). We treated flank and brain tumors with nanocarrier alone without paclitaxel (“Control”) or nanocarrier loaded with paclitaxel. The Control treatment did not impede the growth of either flank or intracranial tumors. DLN was effective against flank tumors, almost completely abrogating the growth of these tumors for the duration of the experiment (Fig. 1D). In contrast to the flank tumors, the intracranial tumors showed much less response to the administered DLN. Intracranial tumors continued to grow robustly despite DLN, with a tumor growth delay of less than one week (Fig. 1E). Moreover, there was no significant difference in the overall survival of the animals with intracranial tumors treated with DLN alone compared with control animals.

**Table 1:** 

	DLN: *TAX-Filomicelle*	Doubling Time: τ_2_ = ln2/*r*	Max Size, *A*
Flank U251	−	2.8 days	40x
	+	>40 days	~1x
Intracranial U251	−	2.9 days	120x
	+	3.7 days	120x
**Fits of Fig.1D,E to solution for Logistic growth:** *dN/dt* = *r N* − *(r/A) N^2^* **where** *N* **is Tumor cell number, and all fits have** R^2^ > 0.95.			

This difference in response to DLN according to tumor location was observed even though intracranial and flank implants had similar cellular growth rates and protein expression profiles. We confirmed that both flank and intracranial tumors contained highly similar protein expression via a novel mass spectrometry analysis algorithm that selects and quantifies only tryptic peptides derived specifically from the human proteome [48]. Two-fold or larger differences in human protein abundance between flank tumors and brain tumors were very uncommon, found in only 5 of 162 human proteins, or 3.1% of the detectable proteome. Specifically, the flank tumors were found to have more extracellular matrix, adhesion, and cytoskeletal proteins, which is not surprising given the collagenous nature of subcutaneous sites (Suppl. Fig. 6).

These results, taken together, suggest that DLN is effective against GBM cells both in culture and when these cells are grown as flank tumors, but DLN is ineffective against the same tumor when it is located within the brain, probably due to the limited permeability of the DLN through the tumor-BBB.

### Targeted radiation therapy disrupts the blood-brain barrier of the brain tumor

MRI studies in patients have suggested that RT can disrupt tumor-associated BBB integrity [21]. To confirm this effect in a mouse-based brain tumor model, we assayed changes in the integrity of the BBB using two established and complementary measures of BBB integrity, the extravasation of Evans Blue (EB) dye and immunoglobulin G (IgG) [39, 40]. EB can be readily visualized under ambient light or fluorescence with high signal intensity over background (Suppl. Fig. 7), but EB must be infused into the animal immediately prior to sacrifice. In contrast, IgG is a normal constituent of serum and is not normally detected in large amounts in the brain. Consequently, the detection of high levels of IgG in the brain indicates extravasation from the systemic circulation through defects in the BBB.

**Figure 2:**
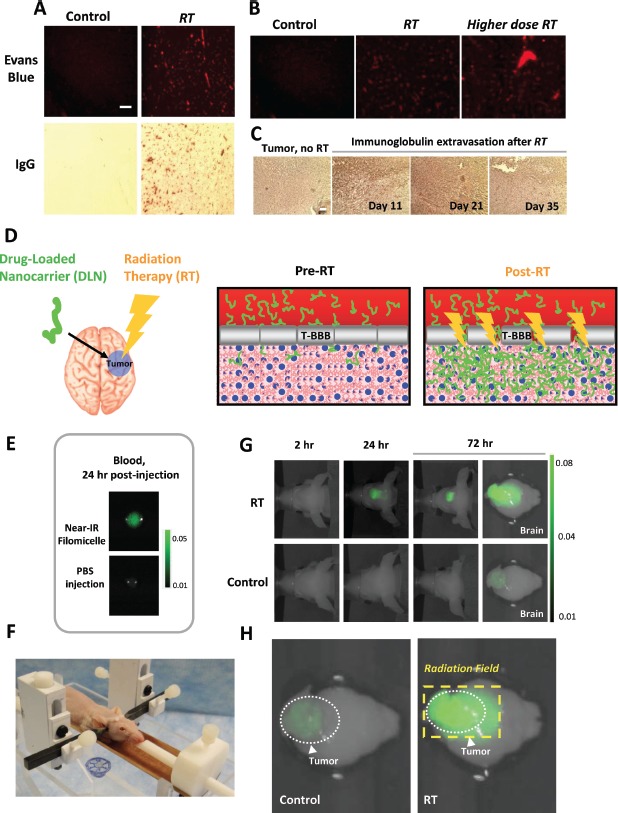
Targeted radiation therapy disrupts the Brain Tumor's blood-brain barrier and permits greater penetration of filomicelle nanocarrier (A) Healthy nude mice were either mock-irradiated (“Control”, left column) or irradiated with 20 Gy in a single fraction to the right cerebral hemisphere (“RT”, right column). Twenty-four hours later, the mice were then injected with Evans Blue dye (EB) into the tail vein (top row), or perfused with 10% formalin via transcardiac injection followed by staining for immunoglobulin IgG extravasation out of the systemic circulation (bottom row). The brains of the mice exposed to RT show substantial extravasation of both EB and IgG while mock irradiated brains show almost no detectable extravasation; differences in area of staining were statistically significant (*p*<0.05). (B) BBB disruption by targeted RT shows a dose-response. Mice were mock-irradiated (left panel) or irradiated with 3 Gy daily × 4 days (middle panel) or 4 Gy daily × 4 days (right panel) and then injected with EB and sacrificed 24 hours after the final radiation fraction and their brains imaged with fluorescence imaging. (C) RT induces Tumor-BBB disruption in GBM intracranial orthografts compared to mock-irradiated tumor-bearing controls. Mice with intracranial GBM orthografts as confirmed by bioluminescence imaging were either mock-irradiated or received cranial irradiation to 3 Gy daily × 4 days. After the indicated time periods following completion of RT, mice were perfused with 10% formalin followed by sectioning and imaging of the brains under microscopy for IgG extravasation out of the systemic circulation. Increased IgG extravasation was evident at 11 and 21 days after RT compared to the un-irradiated controls but was muted by 35 days after RT. The IgG is detected as dark brown regions of staining, representing binding and activity of anti-Ig antibody conjugated to horseradish peroxidase. (D) Therapeutic strategy of leveraging targeted RT-induced BBB disruption to increase DLN accumulation in the brain tumor (tumor denoted in blue; RT denoted by the lightning bolt). (E) Dye-loaded nanocarrier injected into mice is detectable via fluorescent imaging. 100 microliters of blood were withdrawn from mice injected with either nanocarrier loaded with DiR, a hydrophobic near-infrared fluorescent dye (“Near-IR Filomicelle”, DiR) or phosphate-buffered saline (“PBS”), plated onto glass cover slides, and imaged via fluorescent microscopy. (F) Cranial RT was delivered using a novel mouse restrainer and the Small Animal Radiation Research Platform (SARRP). (G) Cranial RT was associated with increased extravasation of dye-loaded nanocarrier into brain tumors. Mice with intracranial tumors of comparable sizes (confirmed by tumor BLI signal) were irradiated (“RT”, 3 Gy daily × 4 days) or mock irradiated (“Control”). *In vivo* fluorescent imaging performed serially after completion of RT revealed significantly increased fluorescent signal in the irradiated brain tumors at 24 and 72 hours compared to the mock irradiated brain tumors (*p*<0.05), indicating increased tumor-BBB disruption induced by radiation. (H) Increased BBB disruption within the brain tumor corresponded to the radiation therapy field. Higher magnification images show that targeted radiation therapy (RT) considerably enhanced BBB permeability beyond that pre-existing within regions of the brain tumor. The dashed box delineates the RT field, within which extravasation of dye-loaded nanocarrier was maximal.

The extravasation of EB and IgG after irradiation of the brain is shown in Fig. 2A. The right cerebral hemispheres of healthy nude mice were irradiated with 20 Gy in a single fraction (a dose employed in stereotactic hypofractionated radiation therapy), followed by assessment for EB and IgG extravasation 24 hours later. The irradiated brains showed substantial EB and IgG extravasation, while mock-irradiated control brains showed no extravasation. Fractionated RT also produced an increase in EB extravasation, with more extravasation seen at higher doses (Fig. 2B).

We next investigated whether RT also affects the BBB within brain tumors. We targeted orthotopic GBM tumors in mice, followed by assessment for IgG extravasation (Fig. 2C). After exposure to RT, brain tumor-bearing regions showed large increases in IgG extravasation, often to a degree greater than that seen in irradiated normal brain tissue (note in Fig. 2C the diffuse, substantial IgG staining in tumor after RT in comparison with the focal peri-vascular IgG staining after radiation in normal brain seen in Fig. 2A).

The increased permeability of the tumor-associated BBB induced by targeted RT was durable. Increased IgG extravasation in irradiated tumors was still very evident at 11 and 21 days after RT, and it was reduced but still detectable by 35 days after RT (Fig. 2C). These results in mice indicate that targeted RT efficiently abrogates the tumor-associated BBB, corroborating previously reported RT-induced modulation of the tumor BBB in patients.

### Radiation therapy increases extravasation of nanocarrier into intracranial tumors

To assess whether the RT-induced increase in brain tumor BBB permeability would facilitate the deposition of larger concentrations of the filomicelle nanocarrier within tumors (Fig. 2D), we administered the nanocarrier loaded not with paclitaxel but with a marker consisting of a hydrophobic, near-infrared fluorescent dye (“DiR”, 1,1'-dioctadecyl-3,3,3',3'Tetramethylindotricarbocyanine iodide) amenable to *in vivo* fluorescent imaging in live mice. We first confirmed that the incorporation of the dye did not affect the half-life of the nanocarrier in the circulation; the nanocarrier-dye assembly remained readily detectable in the blood 24 hours after tail vein injection (Fig. 2E). We then administered the nanocarrier-dye to mice implanted with brain tumors that had undergone fractionated cranial irradiation or mock irradiation. We targeted the anterior portion of the brain containing the tumor using the highly collimated, precise radiation therapy achievable with a novel custom-designed cranial-and body-restraint system in conjunction with the Small Animal Radiation Research Platform (SAARP) (Fig. 2F, Suppl. Fig. 8). These mice subsequently underwent serial *in vivo* fluorescent imaging to detect the dye marker attached to the nanocarrier.

The mice whose brain tumors received targeted cranial irradiation showed more than a five-fold increase above the signal detected in the tumors of mice who were mock-irradiated (*p*<0.05) (Fig. 2G). The increased dye signal appeared in their tumors by 24 hours after irradiation with further intensification by 72 hours. To confirm the accurate targeting of the cranial radiation, and that the radiation increased focal extravasation of the nanocarrier-dye conjugate, the mice were euthanized, and their brains were excised and immediately subjected to fluorescent imaging. Brains containing tumors that were mock-irradiated showed only a small degree of nanocarrier-dye extravasation, presumably due to the presence of sporadic foci of BBB perturbation known to exist within brain tumors (also shown in Suppl. Fig. 7). In contrast, the brains of mice that had undergone SAARP irradiation showed intense extravasation of nanocarrier-dye within the radiation portal that encompassed the brain tumor while showing no increase in fluorescent signal in the unirradiated portions of the brain (Fig. 2H). The increased extravasation of fluorescent dye into brain tumors after targeted RT was therefore consistent with experiments showing similarly increased extravasation of IgG within brain tumors after RT. As an additional test, the fluorescent signal of other tissues and organs from both the brain-irradiated and mock-irradiated mice were compared; these were all found to have similarly low signal intensities that were much smaller than the high signals observed in the irradiated brain and tumor (data not shown). These experiments together indicate that targeted cranial irradiation can significantly increase BBB permeability within brain tumors, leading to significantly greater extravasation and penetration of the filomicelle nanocarrier into the tumors.

### Radiation therapy plus DLN suppresses brain tumor growth and prolongs survival

**Figure 3:**
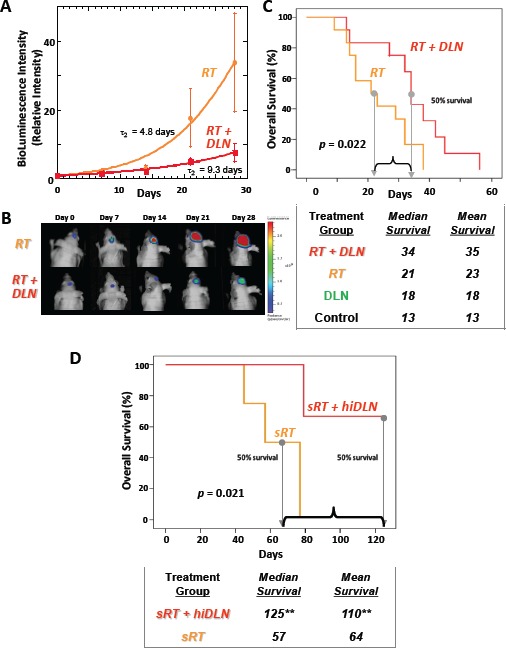
When combined with targeted radiation therapy, DLN has enhanced efficacy against intracranial human GBM tumors and significantly improves overall survival compared to radiation alone Mice with human GBM orthotopic xenografts were treated with targeted radiation therapy alone (“RT”) or in conjunction with DLN incorporating paclitaxel (“RT + DLN”). (A) All the mice then underwent serial bioluminescent imaging (BLI), with values of light emission recorded at least weekly. The graph shows median BLI-fold changes with time from initiation of treatment. The significantly slower growth of tumors treated with RT + DLN demonstrates superior anti-tumor effect compared to RT alone (*p*<0.05). (B) Images show representative BLI of mice with intracranial tumors that received treatment with RT alone or RT + DLN. Both groups of mice underwent serial imaging at the times listed after treatment. (C) Graph showing overall survival of mice with brain tumors that received treatment with RT alone or RT in conjunction with DLN incorporating paclitaxel. The table below the graph lists the median and mean survival of each group of treated mice, along with mice that were treated with DLN alone or mock-treated (Control). In mice with intracranial tumors, combined RT + DLN resulted in significantly improved mean and median survival compared to mice receiving RT alone, DLN alone, or mock treatment (*p*<0.05). (D) Graph showing overall survival of mice with brain tumors that received treatment with stereotactic, optimized radiation therapy techniques alone (“sRT”) or sRT in conjunction with higher dose DLN (“hiDLN”) incorporating paclitaxel. The table below the graph lists the median and mean survival of each group of treated mice. The combined DLN and optimized radiation therapy techniques resulted in significantly improved mean and median survival compared to mice receiving radiation therapy alone (*p*<0.05). (** Most of the mice treated with combined hiDLN and sRT remained alive, in excellent condition, gaining weight, and with no detectable tumor. The median and mean survivals for “sRT + hiDLN” at 125 days following treatment are 125 and 110 days respectively.

We next sought to test the efficacy of nanocarriers loaded with a chemotherapeutic agent in irradiated brain tumors. Mice implanted with brain tumors were treated either with RT alone to the whole brain (3 Gy daily × 4 days) or RT combined with paclitaxel-loaded nanocarrier (RT + DLN). Both RT and RT + DLN were effective in slowing intracranial tumor growth, but the combination treatment was significantly more effective than RT alone as assessed by bioluminescence imaging (*p*<0.05) (Fig. 3A-3B).

We found the DLN was well-tolerated by the mice, even when administered in combination with RT. The most clinically relevant endpoint is survival, which reflects tumor control but is also affected by any potential complications of treatment. Mice treated with the RT + DLN continued to eat and maintain body weight until tumor progression reached a lethal threshold. The mean and median overall survival of mice with intracranial tumors treated with RT + DLN was superior to mice receiving RT alone or DLN alone (mean: 35 vs. 23 vs. 18 days; median: 34 vs. 21 vs. 18 days, respectively, *p*<0.001 for all comparisons) (Fig. 3C, Suppl. Fig. 9). The addition of RT to DLN treatment therefore resulted in an improvement in median survival of >50% compared to animals that received RT or DLN alone. Furthermore, mice treated with combined RT and DLN successfully maintained body weight (Suppl. Tables 2-3). These experiments together indicate that the combination of DLN with targeted cranial radiation results in improved survival.

While these results were encouraging, we investigated whether the efficacy of combined modality therapy could be further improved by increasing the dose of paclitaxel incorporated into the nanocarrier and simultaneously optimizing radiation treatment techniques to a level of sophistication more closely resembling that which is administered to patients. Brain tumors are often treated with stereotactic techniques in which the tumor is delineated via pretreatment imaging, and fiducial markers or specialized hardware are used to mark the location of the tumor to ensure the highest degree of accuracy and reproducibility in targeting with RT. We therefore constructed for the mice a version of head-frames employed for stereotactic radiation therapy consisting of a plastic bed with Teflon screws that insert into the external acoustic meatus of the mouse. This novel, non-traumatic restraint simultaneously minimized head rotation and maximized positional reproducibility (Fig. 2F, Supp. Fig. 8) [34]. This setup integrated into the SARRP and allowed us to perform cone-beam CT for target delineation before each radiation therapy administration [49]. These techniques together enabled us to target brain tumors with a high degree of accuracy and reproducibility. We coupled these more sophisticated RT treatments with the use of hiDLN, our preparation of the nanocarrier that delivered 48% of the daily maximum tolerated dose (MTD) of intraperitoneal (IP) paclitaxel [43], or 9.55 mg/kg, given as 1 mL IP treatments, rather than the 29% of MTD delivered in earlier experiments. We found that the optimized radiation therapy techniques, even with the higher dose of paclitaxel, were well-tolerated by the mice. Many of the treated mice gained weight during treatment compared to their pre-treatment weight, presumably because of tumor regression and because of reduced toxicity from radiation therapy as improved radiation therapy targeting resulted in greater sparing of normal tissues such as the oropharyngeal mucosa.

By optimizing both radiation therapy techniques and DLN paclitaxel dosing, we achieved the greatest overall effect. This combination of radiation therapy and hiDLN now resulted in median survivals that were more than double the survivals of animals receiving RT alone (*p*<0.05) (Fig. 3D). Moreover, a majority of mice treated with the combined (RT+hiDLN) treatment remained alive and thriving at the end of the experiment with no evidence of residual brain tumor. In contrast, all mice treated with RT alone died. These results together support the efficacy of combined modality therapy using targeted radiation therapy to disrupt the tumor BBB to facilitate greater DLN penetration into tumors, resulting in improved treatment efficacy compared to the results achieved with either RT or DLN alone.

## DISCUSSION

The treatment of intracranial tumors such as GBM or brain metastases remains a particularly difficult therapeutic challenge. The BBB prevents accumulation of potentially harmful chemicals, such as chemotherapy, within the central nervous system and therefore helps protect against adverse effects on cognition and other critical functions. The portion of the BBB that is within intracranial tumors likewise impedes the accumulation of therapeutic levels of chemotherapy and attempts to identify new chemotherapeutic agents that can effectively penetrate the tumor-BBB are still ongoing [16, 50, 51]. A recent animal study of experimental brain metastases from human breast cancer cells found that although most metastases showed some degree of tumor BBB permeability compromise, the concentrations of paclitaxel or doxorubicin accumulated within these brain metastases were <15% of that achieved in extracranial metastases [52]. Consequently, the tumor BBB impedes the accumulation of therapeutic concentrations of both of these agents thereby limiting their effectiveness for treating intracranial tumors.

The presence of a structural barrier within the brain impeding the accumulation of therapeutic levels of drug likely helps explain why chemotherapy agents such as paclitaxel, which are lethal against GBM cells *in vitro*, fail to improve outcomes in patients with brain malignancies when tested in clinical trials [4]. Our results, as well as those of Lockman et al., indicate that although the vasculature within an intracranial tumor is partially permeable to macromolecules as evidenced by limited extravasation of IgG in orthotopic tumor xenografts [52], this partial permeability is insufficient to allow accumulation of therapeutic levels of drug. We therefore pursued a dual approach towards improving therapeutic efficacy: using a drug delivery vehicle that enhances circulation time and drug retention in tumors while using radiation to increase the permeability of the tumor BBB to the chemotherapeutic agent.

Nanopolymer therapeutics represent a novel and expanding class of drug delivery systems that are currently showing great promise. Such agents can often be administered intravenously or intraperitoneally and can incorporate many anticancer drugs. Customized configurations of shape, size, and composition of the nanocarrier can substantially influence the *in vivo* performance of the therapeutics. The OCLA developed here is a new PEG-based *block* copolymer that randomizes lactic acid and caprolactone in the hydrophobic chain (Fig. 1A) to minimize pure polyester crystallinity and glassiness [41] that, in turn, tend to limit drug loading and make the filomicelles more rigid. Flexibility fosters circulation of filomicelles for days longer than smaller, more spherical assemblies of the same composition [29]. This increased serum half-life facilitates diffusion of the drug-loaded carrier into solid extracranial tumors that lack a permeability barrier analogous to the blood brain barrier while enhancing drug retention within the tumors to increase treatment efficacy. The blood brain barrier effectively limits the enhanced permeability and retention (EPR) effect, explaining why DLN that are effective against extracranial tumors are ineffective against intracranial tumors. For this series of experiments, we chose a microns-long filomicelle nanocarrier whose flexibility increases serum half-life but whose larger size makes it even less likely to permeate through an intact blood brain barrier than smaller nanocarriers so that any increased extravasation of DLN observed after RT can be ascribed to disruption of BBB permeability by RT.

We chose an orthotopic U251 tumor model for these studies because in our experience, and in the experience of other labs, this model recapitulates many of the key features of clinical GBM, including rapid growth, necrosis, hypoxia, increased VEGF expression, and invasive growth [33-37], although we acknowledge that any implantable mouse model for GBM has inherent limitations. We considered using an endogenous GBM model, but we felt that the orthotopic model had distinct advantages because it allowed us to grow tumors of consistent size and anatomic location so that we could stratify mice evenly to different treatment groups and deliver the same focal, stereotactic radiation to each tumor. The heterogeneous anatomic locations and different sizes of endogenous tumors would have been confounding factors for our survival studies. Our novel technique for precise, image-guided treatment of mouse brain tumors using the Small Animal Radiation Research Platform (SARRP) micro-irradiator and our home-built stereotactic restrainer, an approach closer to clinical radiation treatment methods than previously reported in the mouse literature [34], required reproducible tumors to ensure that radiation treatment plans would be identical so that RT toxicity and integral dose to critical brain structures would be equivalent across all treatment stratifications.

We specifically chose paclitaxel to load in the nanocarrier rather than temozolomide, the current treatment of choice for clinical GBM, because there is evidence that paclitaxel is more potent *in vitro* against GBM [3] and because in its free form it has relatively poor penetration of the tumor BBB even when combined with radiation, accounting for the disappointing outcomes in a Phase II clinical trial of RT + paclitaxel vs. RT alone for GBM [4]. Testing a drug like paclitaxel that has poor tumor BBB penetration in its free form, even with RT, allowed us to conclude that any treatment benefit was likely derived from enhanced delivery of paclitaxel across the tumor BBB as a result of the nanocarrier, rather than dissociation of the drug from the nanocarrier or degradation of the nanocarrier.

We employed the filomicelle DLN loaded with paclitaxel that previous work has shown to enhance the duration of systemic circulation and EPR. In previous experiments, the empty nanocarrier was very well tolerated with no ill effects observed in nude mice and no increase in apoptosis observed in xenograft tumors or in key normal tissues like the heart, lung, liver, spleen, and kidney compared to mice injected with equivalent volumes of normal saline [11]. There was also no anti-tumor treatment effect observed for the empty nanocarrier [11]. The nanocarrier loaded with paclitaxel had a significantly higher MTD than free paclitaxel in nude mice and demonstrated greater tumor specificity with less apoptosis observed in normal organs compared to free paclitaxel [11]. This agent proved to be safe in our experiments with almost no discernible toxicity noted in treated animals despite daily administration of amounts approaching levels at which 50% of animals treated with free paclitaxel alone would be expected to show morbidity.

Compared to the efficacy of paclitaxel-loaded DLN for treating extracranial tumors, we found that paclitaxel-loaded DLN had insufficient effect on intracranial orthotopic tumors to be considered as a treatment for GBM or metastatic disease. Mice treated with the drug-loaded nanocarrier had only a minimal, transient reduction in the growth of their intracranial tumors and only a modestly improved survival compared to control animals receiving nanocarrier without paclitaxel. These observations suggested that the DLN could not, by itself, effectively penetrate through the intracranial tumor BBB.

Prior work has suggested that radiation therapy could disrupt the BBB, including the tumor BBB [21]. We hypothesized that radiation-induced disruption of the tumor BBB might enhance permeation and retention of vascular-delivered therapeutic reagents within the targeted tumor. We confirmed that this was indeed the case, through both *in vivo* and *ex vivo* experiments. Radiation therapy (RT) by itself disrupted the tumor BBB at doses that were well-tolerated by the animals as shown by the increased extravasation of fluorescent dye-loaded nanocarrier into brain tumors following RT. In our experiments, mice treated with RT + dye-loaded nanocarrier had equivalent outcomes to those treated with RT alone, mirroring the findings of Vinchon-Petit et al. that the empty nanocarrier has very low toxicity and no intrinsic treatment effect [53].

We subsequently showed that the combination of RT and paclitaxel-loaded DLN resulted in a therapeutic response that was significantly better than the results achieved with either RT or paclitaxel-loaded DLN alone. In our preclinical study, the median overall survival of subjects treated with the combination of RT + DLN was improved over RT alone by 13 days. This extended survival represents an increase over RT alone of approximately 61%. To put this into the appropriate clinical context, temozolomide, the most important new drug to enter the armamentarium against GBM in the last 10 years, when combined with radiation results in an increase in survival duration of only ~21% over RT alone in GBM patients [2]. When we optimized our radiation techniques to enhance conformality of dose delivery with the tumor target and we increased the dose of paclitaxel integrated into the DLN, we observed additional improvements in survival. A majority of the animals treated with optimized radiation therapy techniques and hiDLN remained alive at the end of the experiment with no detectable signs of tumor. We further note that the animals tolerated this combination of targeted RT and hiDLN remarkably well. Despite the presence of advanced tumors, all mice tolerated repeated administrations of DLN without discernible toxicities or weight loss.

These results together indicate that targeted RT can usefully serve as a modulator of tumor BBB permeability to enhance therapeutic drug delivery to intracranial tumors such as GBM. Furthermore, the increased serum half-life and EPR properties of DLN result in enhanced accumulation of therapeutic anticancer drugs within the irradiated intracranial tumor once the tumor BBB is breached. While clinical trials would clearly be required to establish the safety of DLN formulations in patients when combined with radiation, these observations are encouraging. The success of DLN combined with RT against bulky, non-resected tumors in mice suggests that similar therapeutic approaches might have a future role in the treatment of intracranial neoplasms such as GBM whose prognosis is notoriously grim with current treatment options. The outcome of such clinical trials to test the efficacy and safety of exciting new agents that include drug-loaded nanocarriers would be eagerly awaited by patients and healthcare providers alike.

## MATERIALS AND METHODS

All experiments with animals were performed in accordance with IACUC guidelines according to an IACUC-approved protocol, and all surgical procedures were conducted according to current institutional guidelines for animal experiments.

### Modification of Tumor Cells for Stable Expression of Firefly Luciferase & GFP

The human-derived GBM tumor cell line U251 was transduced with a lentiviral construct (pGreenFire, purchased from System Biosciences) containing the firefly luciferase gene and the green fluorescent protein (GFP) gene under the control of an SFFV (spleen focus-forming virus) promoter. 200,000 cells were plated and washed with PBS. Thawed viral particles in 250 μL Dulbecco's Modification of Eagle's medium (DMEM) were added to the cells. Cells were allowed to incubate for 72 hours. After transfection, supernatant with virus was collected and passed through a 22 nm filter. The filtered supernatant was used to infect the target U251 cells. The transduced U251 cells were sorted twice with flow cytometry to select the top 1% of cells based on fluorescence for propagation in culture and for implantation as subcutaneous or intracranial xenograft tumors in nude mice.

### *In vitro* Analysis of Tumor Cell Luminescence

Transduced cells were trypsinized, washed with PBS, counted with a Coulter counter, and plated at halving dilutions in the wells of a 24-well plate. An identical corresponding dilution series of unmodified cells was plated as a negative control. Four hours after plating, a saturating dose of D-luciferin was added to each cell sample, and luminescent signals were recorded using the IVIS Lumina system.

### Polymer Synthesis and Preparation of Filomicelle Paclitaxel-loaded Nanocarrier

The flexible filomicelles consist of a degradable di*block* copolymer that is synthesized by ring-opening polymerization of the hydrophilic poly(ethylene oxide) ε-caprolactone using D,L-lactide and methoxy terminated poly(ethylene oxide) as a macro initiator and stannous octoate as catalyst. Briefly, freshly distilled ε-caprolactone (2.5 g, 0.0219 mol), 2000g/mol of poly(ethylene oxide) (PEO), D,L-lactide, and stannous octoate (15 mg, 3.7 × 10-5 moles) were weighed out in a flamed and nitrogen-dried ampule. The ampule was sealed and placed in an oven pre-equilibrated to 140°C, and the polymerization reaction was allowed to proceed for 4 hours. The reaction was terminated after cooling the ampule to room temperature.

A solvent evaporation method was then used to generate the filomicelles, which were combined with paclitaxel in methanol, dialyzed against PBS and filtered by extrusion to remove any paclitaxel aggregates to thus generate the drug-loaded nanocarrier formulation. For preparation of our standard concentration of DLN, the polymer concentration was 4 mg/ml and paclitaxel was added to achieve polymer/paclitaxel weight-to-weight (w/w) ratio of 20:1. We also prepared a more concentrated nanocarrier-paclitaxel solution (HiDLN) starting with a polymer concentration of 20 mg/ml with paclitaxel added to achieve a w/w ratio of 32:1. Increased addition of paclitaxel does not lead to better loading because of precipitation of polymer and paclitaxel.

Detailed information on the synthesis, purification, and analysis of the paclitaxel-loaded nanocarrier is provided in the Supplemental Section and can also be found in previous publications [11, 41].

### Intracranial Implantation of Tumor cells

We used an orthotopic model system to ensure that the mice in the different treatment groups had similar brain tumors. All tumors were implanted into the right side of the brain, were sufficiently large to approximate the tumor burden observed in GBM patients without being so large that the animals would expire before treatment effects could be ascertained, and were placed in a location and limited to a size that could be precisely radiated using the image-guided Small Animal Radiation Research Platform micro-irradiator (SARRP). The uniformity of the brain tumors was designed to ensure that RT toxicity and the integral dose to critical brain structures would be equivalent in the various treatment groups [34, 42].

Nude athymic NCr mice (nu/nu) at 6 weeks of age were obtained from the NCI. One hour prior to intracranial implantation the mice were weighed and pre-medicated with a subcutaneous injection of 5 mg/ kg of meloxicam in saline to control for post-operative pain and inflammation. The mice were anesthetized with an IP injection of ketamine/xylazine at doses of 140mg/kg and 10mg/kg respectively. Once the anesthesia extinguished the mouse's response to light pinching of the rear foot, the mouse was positioned in a stereotactic device (Stoelting Digital Just for Mice Stereotactic Instrument) for improved immobilization of the animal and to enhance the precision of the intracranial implantation. The mouse's temperature was monitored throughout the surgery with a rectal probe and maintained at 37°C with feedback control to a heating plate positioned underneath the animal. An ophthalmic ointment was applied to the eyes to prevent drying. Using aseptic technique, the top of the head was swabbed with betadine, and a small, 0.75 cm longitudinal incision was made through the scalp to expose the skull. Using the stereotactic armature, a small hole was drilled using the Foredom Microdrill with a 0.45 mm burr at the injection site at coordinates 2 mm posterior and 1.5 mm lateral to the bregma in the right cerebral hemisphere. Through this aperture, a stereotactically-guided syringe with a 30 gauge flat bevel needle (Hamilton Syringe) delivered in sterile fashion approximately 300,000 luciferase/GFP-expressing U251 human-derived brain tumor cells in 6 microliters of DMEM media 2.5 mm deep within the brain parenchyma at an injection rate of 0.5 μL/minute using the Automated Injector Pump by Harvard Apparatus [42]. After injection, the needle was left in the brain for a period of 2 minutes and then slowly withdrawn. The incision was closed with bone wax, the skin re-approximated with glue, and the animal was moved to a heated pad for the post-operative recovery.

### Flank Implantation of Tumor cells

Flank injections of U251 cells identical to the cells used for the intracranial injections were performed. Mice were injected subcutaneously with tumor cells at a site superficial to the scapulae bilaterally and superficial and medial to the hip joints bilaterally. The mice were anesthetized with an IP injection of ketamine/xylazine at doses of 140mg/kg and 10mg/kg respectively and then placed on their abdomen with their limbs symmetrically arranged such that their feet were pointed cephalad. Once a mouse was sufficiently anesthetized so that no response was elicited from a light pinch of the rear foot, the site of injection was cleaned with alcohol. Using aseptic technique, an injection of 100 microliters of a cell suspension containing 2 × 106 tumor cells along with DMEM media was administered using a 26 gauge 5/8” needle into each site. No closure was necessary.

### Radiation Therapy

For each control and treatment group, mice were evenly divided based on pre-treatment weight and maximum bioluminescent signal intensity. Mice were anesthetized with ketamine/xylazine at doses of 140 mg/kg and 10mg/kg respectively, and then individually mock-irradiated or irradiated in either a Mark I cesium irradiator (J. L. Shepherd, San Fernando, CA) or the Small Animal Radiation Research Platform (SARRP) (Xstrahl, Surrey, United Kingdom), at a dose rate of 0.1 Gy/min. For mice irradiated using the cesium irradiator, each mouse was fitted with a lead shield that exposed only the desired target before being placed in the irradiator. Radiation was administered with a unidirectional beam for a pre-determined length of time corresponding to the desired dosage. Unless otherwise stated, the radiation dose was 3 Gy/fraction administered once daily for four days.

Mice treated with optimized radiation techniques were treated in a custom-made plastic mouse bed equipped with a stereotactic head immobilizer that integrated with the SARRP [34]. The anatomy of the skull and brain was imaged with the SARRP's onboard cone-beam CT. The location of the tumor was determined by bioluminescent imaging, and the skull was tattooed to define the beam's location. All these techniques helped ensure day-to-day reproducibility and precise delivery of the radiation therapy [34].

In experiments to test the efficacy of paclitaxel-loaded nanobiopolymer combined with fractionated radiation therapy, the standard concentration of DLN was given as a 1 mL intraperitoneal (IP) injection every other day for 17 days beginning on day 1 of radiation. Mice in the RT alone group received IP injections of phosphate-buffered saline (PBS) of equal volume. Mice received up to 29% of the daily maximum tolerated dose (MTD) of IP paclitaxel [43], or 5.89 mg/kg, per treatment. This dose was approximately equivalent to 26% of the human MTD as determined in a Phase II trial of GBM using concurrent RT and free paclitaxel [4]. Overall survival was calculated based on death or sustained loss of >20% of pre-treatment weight, an IUCAC guideline for mandated euthanasia. Kaplan Meier survival analysis was performed using the SPSS Software program.

A second experiment tested the efficacy of RT+hiDLN vs. RT alone against intracranial tumors. Mice in the RT+hiDLN group were given 1 mL IP injections of the more concentrated hiDLN every other day for 34 days (17 total treatments) beginning on day 1 of radiation. Mice in the radiation alone group received IP injections of PBS of equal volume. RT+hiDLN mice received 48% of the daily MTD of intraperitoneal paclitaxel, or 9.55 mg/kg per treatment. This dose was roughly equivalent to 42% of the human MTD from a study of GBM patients undergoing concurrent treatment with free paclitaxel and RT [4].

### Bioluminescent Imaging

Mice were imaged using the IVIS Lumina II bioluminescence imaging system beginning 1 week after tumor injection. The mice were anesthetized by IP injection with ketamine/xylazine (140mg/kg / 10mg/kg). Once fully anesthetized, the mice received a subcutaneous injection of 60 microliters of pharmaceutical-grade D-luciferin (50 mg/mL) which freely crossed the BBB [44]. Imaging via the IVIS system commenced at 5 minutes after the luciferin injection was delivered. The mice were repeatedly imaged over a span of 30 minutes to determine the maximum luminescence intensity in photons/second, a surrogate marker for the size of the tumor(s). IVIS imaging was repeated at least weekly to measure tumor size. All bioimaging studies followed the IACUC-approved guidelines for the Optical Imaging Facility. After imaging acquisition, a volume of interest (VOI) was drawn around each tumor and a background VOI was drawn on the animal in an area known to be free of tumor to generate a background-corrected bioluminescence flux value for each tumor at each given time point. The maximum background-corrected value for that tumor during the 30 minute imaging session was used as the maximum bioluminescent value. The bioluminescence of tumors was tracked over time using a measure termed “fold”, which was defined as: [current max BLI signal] / [initial pre-treatment max BLI signal] [45, 46].

### Assays of Blood-Brain Barrier Integrity

Blood-brain integrity was assessed by extravasation of immunoglobulin G (IgG) or Evans Blue dye (EB), a large molecular weight (961 Da) dye [39, 40]. For EB assays, mice were anesthetized with ketamine/xylazine and then given a tail vein injection of 2% Evans Blue dye mixed in 1xPBS (4 ml/kg body weight, Sigma, E2129) using a 26G 5/8” needle. The Evans Blue was allowed to circulate for 5 hours before the mouse was re-anesthetized with ketamine/xylazine. The circulating un-extravasated EB was then removed by continuous perfusion. This was accomplished by opening the chest cavity and exposing the heart. An 18 gauge needle was carefully inserted into the left ventricle, and PBS was continuously infused. A separate incision was made in the right atrium to allow drainage of blood and replacement with 1xPBS. The infusion was continued until the circulating fluid was clear. After perfusion, the whole brain tissue was dissected, removed, and post-fixed in 10% formalin overnight. The following morning, the brain was placed in 30% sucrose for 3 days, and transferred to isopentane until frozen (stored in a -80°C freezer). Coronal tissue was sectioned at 20 μm intervals slices with a cryostat (Microm HM 505 E), stained, and photographed using light microscopy and fluorescence microscopy set to the red channel for Evans Blue visualization. Immunostaining for the extravasation of IgG immunoglobulin as a measure of BBB permeability was performed by opening the mouse's chest cavity and pumping a continuous infusion of 1xPBS into the left ventricle through an 18 gauge needle as described above, followed by perfusion with 10% neutral buffered formalin. The brain was removed and processed as above. Sections were stained with anti-mouse IgG antibodies (1:200 concentration), using horseradish peroxidase-streptavidin (1:400 concentration) and diaminobenzidine for visualization. Stained sections were imaged using light microscopy.

### DiR near-infrared dye-loaded nanocarrier experiments

To determine if RT facilitated extravasation of the nanocarrier across the tumor BBB, we *linked* the nanocarrier to DiR, a near-infrared fluorescent dye with absorption of 748 nm and emission of 780 nm, using the same protocol used for encapsulating paclitaxel in the nanocarrier. Mice implanted intracranially with U251-GFP-Luc cells according to our protocol were stratified according to their BLI tumor signal intensity and randomized to receive either mock irradiation (n=3) or focal irradiation to their tumor implants to a dose of 12 Gy in 4 daily fractions of 3 Gy/fraction (n=4). 10 days after RT, the mice were injected intravenously via tail vein with 250 μL of the dye-loaded nanocarrier solution. A third group of previously untreated mice without tumors received an equal volume of PBS via tail vein injection while a fourth group of previously untreated mice without tumors received an equal volume of the dye-loaded nanocarrier. *In vivo* and *ex vivo* near-infrared fluorescence imaging of the brains of all of these mice was performed using the LiCor Pearl imaging system using the 800 nm wavelength setting. Mice were anesthetized in the usual manner prior to imaging. *In vivo* brain imaging was performed at 24 hours, 48 hours, and 72 hours after injection of the dye-loaded nanocarrier or PBS. At 72 hours after injection, the brains were harvested, washed in PBS, and imaged *ex vivo* using the LiCor Pearl. Fluorescent signal intensity was assessed quantitatively by comparing the activity in a background region of interest (ROI) to the signal intensity from an equal sized region of interest within the brain.

### FUNDING

National Institutes of Health (RC1 CA145075 to G.K., R01 EB007049 to D.D., K08 NS076548-01 to J.D.).

University of Pennsylvania Nano/Bio Interface Center (NBIC)

## Supplementary Text, Figures and Tables




