# Supplementary material for: Enhancing the Efficacy of Drug-loaded Nanocarriers against Brain Tumors by Targeted Radiation Therapy
Source: Oncotarget. 2013 Dec 23;4(1):64–79. doi: 10.18632/oncotarget.777 (PMC3702208; doi:10.18632/oncotarget.777)
Supplement: Supplementary file 1 [file oncotarget-04-064-s001.doc]

Enhancing the Efficacy of Drug-loaded Nanocarriers against Brain Tumors by Targeted Radiation Therapy-Baumann et al

**SUPPLEMENTAL TEXT**

***Materials***

ε-caprolactone was purchased from Alfa Aesar (Ward Hill, USA); methoxy poly(ethylene oxide) (2000 g/mole) (MePEO), and fluorescent PKH26 dye were purchased from Sigma (St. Louis, MO). Stannous octoate was purchased from MP Biomedicals Inc., Germany. Paclitaxel was purchased from LC laboratories (USA). All organic solvents were analytical grade from Fisher Scientific. Fluorescent near IR dye, DiR, was purchased from Invitrogen Inc. (USA).

***Synthesis and characterization of diblock copolymer****.*

Amphiphilic di-block copolymer denoted as OCLA corresponds to poly(ethylene glycol)-*block*-poly(ε-caprolactone-*random*-*D,L*-lactide) (PEG_45_-*b*-P(CL_92_-*r*-LA_21_), where the subscript for each polymer block indicates the degree of polymerization of respective blocks. In terms of molecular weights, PEG MW is 2000 and the polyester block is 12,000 g/mol, denoted as OCLA [11, 41]. Copolymers were synthesized by standard ring opening polymerization of ε-caprolactone and *D,L*-lactide monomer using MePEO (2000 g/mol) as initiator and stannous octoate as catalyst. Briefly, freshly distilled ε-caprolactone (2.19 g, 1.92 x 10^-2^ M), *D,L* -lactide (0.312 g, 2.15 x 10^-3^ M ) and Me-PEO (2000 g/mol) (0.4166 g, 2.17 x 10^-5^ M) calculated based on the desired PEO_45_-*b*-P(CL_92_-*r*-LA_21_) block ratio and stannous octoate (15 mg, 3.7 x 10^-5^ M) were taken in a previously flamed, argon-purged glass ampule (10 mL). The ampule with reactants was further argon-purged for a few minutes, vacuumed for 15-20 minutes to eliminate the trace amount of moisture and sealed under vacuum. The polymerization reaction was allowed to proceed for 6 hours at 140 °C in oven. The reaction was terminated after cooling the ampule to room temperature.

The newly synthesized OCLA polymer was assessed from ^1^H NMR spectra obtained from a Bruker NMR360 spectrometer using deuterated chloroform (CDCl_3_) as solvent. The molecular weight distribution of the synthesized polymer was determined by gel permeation chromatography using a Waters GPC system equipped with a Waters 1215 binary pump and Waters 2414 refractive-index detector. Separation was performed using Styragel HR2 column, calibrated with polystyrene standards and tetrahydrofuran as solvent.

***Preparation of filomicelle from OCLA polymer and Fluorescence microscopy imaging***

Filomicelles (4 mL) was prepared from OCLA (2, 12) polymer by solvent evaporation method as described previously. Briefly, OCLA polymer (1.43 x10^-3^ mM) were dissolved in 250 µL of chloroform and the prepared polymer solutions were added to 4 mL of DI water in a clean 15 mL flat bottom glass vial. Micellization was induced by slow evaporation of chloroform by stirring the mixture at room temperature under gentle speed (~110 rpm). The stirring was continued for 60 hours for complete removal of chloroform. For fluorescent imaging of the resultant filomicelles, 50 µL of polymeric micelle solution (100 µM) was taken into an Eppendorf tube and labeled with 0.2 µL of 0.2 mM hydrophobic fluorescent dye (PKH26, Sigma). An aliquot of 2 µL of dye -labeled micelle sample was placed on a microscope slide and covered with a round cover-slip (18 mm × 1mm), pressed gently and sealed with vacuum grease on the edges. The micelle sample was imaged using a 60X lens with oil on Olympus IX71 microscope equipped with a Cascade 512B camera.

***Contour length distribution of worm micelles***

An aliquot of 50 µL of fluorescently labeled worm micelle solution (100 µM) was diluted to 400 µL with DI water in an Eppendorf tube. 10 µL of NaCl solution (100 mM) was added to the worm solution and mixed gently. Salt mixed 3 µL of diluted worm solution was dropped on a microscope cover-slip (diameter: 18 mm), pressed hard to make a thick film, sealed the slide with vacuum grease and imaged by an Olympus IX71 microscope using 60x object equipped with a CCD camera. The addition of salt immobilizes and sticks the individual worm micelle to the glass surface facilitating the measurement of worm contour length. For each sample, few hundreds of worm from 10/12 frames were measured using image J program.

***Paclitaxel incorporation into Worm-like Filomicelles - preparation of DLN***

Paclitaxel was solubilized in methanol (50 mg/mL) and 50 µL of Paclitaxel (2.5 mg) solution in methanol was added to the freshly prepared worm micelle solutions (4 mL) in a glass vial, stirred for 30 min at RT and left overnight with closed cap. Next day, paclitaxel loaded worm micelle formulation was dialyzed (Spectrapor membrane, MWCO: 3500 KD) against DI water for 2 hours to remove methanol and unloaded free paclitaxel and finally against PBS to make the worm solution isotonic for in vivo administration. After dialysis, the sample was transferred to 10 mL polystyrene sterile culture tube and left overnight to settle unassembled polymer and unloaded paclitaxel aggregates. The filomicelle supernatant was separated and transferred to another sterile culture tube for in vivo injection. All transferring of filomicelle solution was performed under sterile condition to maintain the sterility of the formulation.

***HPLC analysis to quantify paclitaxel loading***

A Shimadzu HPLC system (Shimadzu Corporation, Japan) equipped with an inline DGU-20A3 vacuum degasser, LC-20AB binary high pressure pump, SIL-20AC high speed auto sampler and a pinnacle® DB C18 reverse-phase column (4.6 × 150 mm) was used to detect and quantify paclitaxel in worm formulation. Briefly, 20 µL of sample was injected to the HPLC system and eluted by a mobile phase consisting of acetonitrile and water with 0.1% of trifluoroacetic acid at a flow rate of 1 ml/min at 35 °C. In gradient elution, the starting acetonitrile concentration was 50% and increased to 60% within 15 min at a constant rate. The detection was performed at λ=228 nm and quantified using a diode array detector (SPD M20A, Shimadzu). A standard curve was prepared from paclitaxel at concentration of 0.002, 0.01, and 0.05 mg/ml. Data was acquired and processed with LC solution chromatography software from Shimadzu Corporation. Paclitaxel - loaded OCLA worm micelles was mixed with acetonitrile and water at a ratio of 50:50 to break the micelle structure and solubilize the paclitaxel followed by the HPLC analysis using the standard curve described above.
