# Supplementary material for: Enhancing the Efficacy of Drug-loaded Nanocarriers against Brain Tumors by Targeted Radiation Therapy
Source: Oncotarget. 2013 Dec 23;4(1):64–79. doi: 10.18632/oncotarget.777 (PMC3702208; doi:10.18632/oncotarget.777)
Supplement: Supplementary file 2 [file oncotarget-04-064-s002.pdf]

Enhancing the Efficacy of Drug-loaded Nanocarriers against Brain Tumors by Targeted Radiation Therapy - Baumann et al

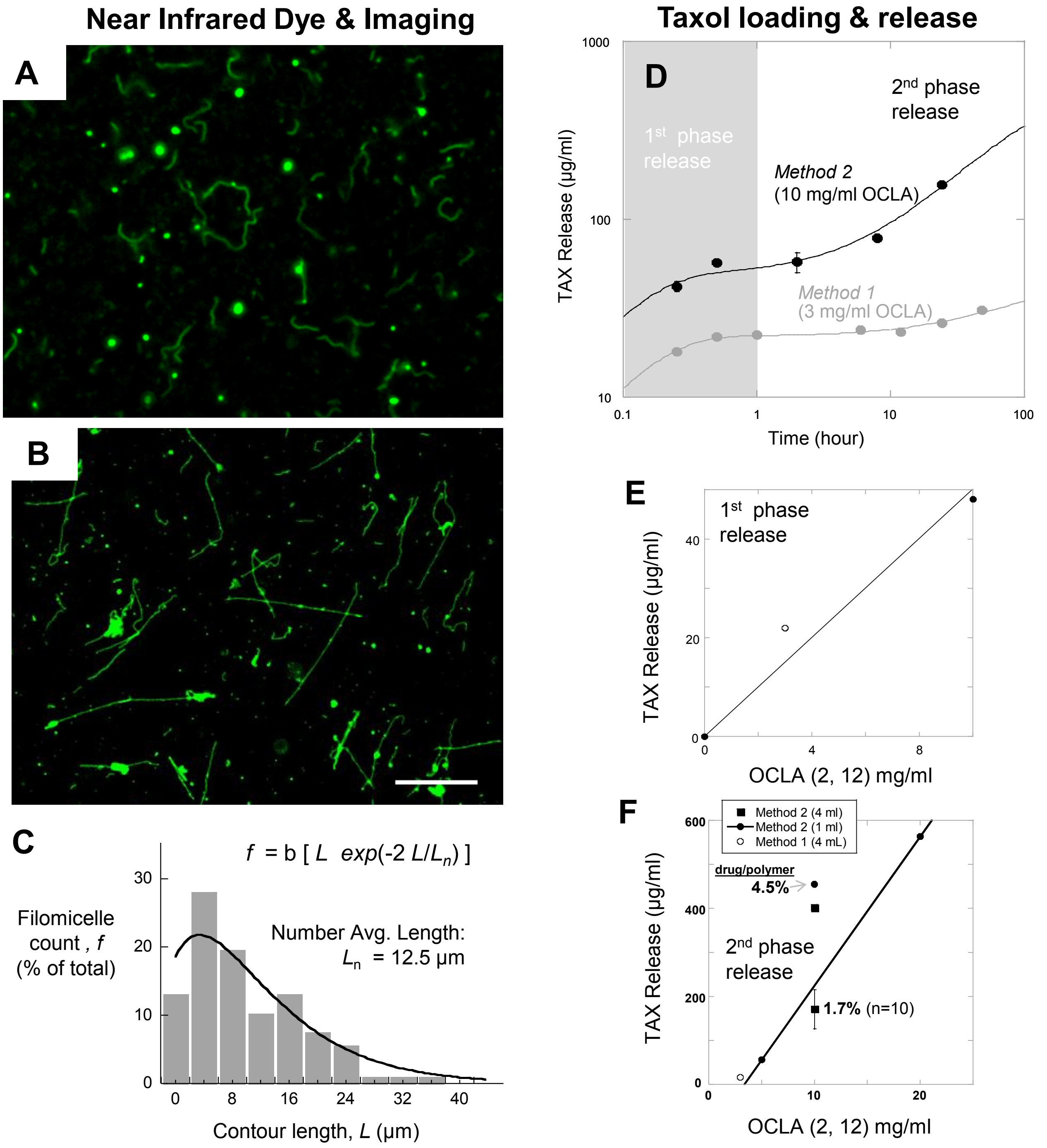

Supplemental Figure 1: Fluorescent dye- and Taxol-loaded worm-like Filomicelles. (A) Representative fluorescence microscopy snapshot of worm-like filomicelles made with OCLA and labeled with fluorescent dye. (B) To measure the mean contour length, filomicelles were immobilized and electrostatically straightened on a microscope slide by adding 7 mM of NaCl. Scale bar 10 µm. (C) Contour length distribution of several hundred OCLA (2, 12) Filomicelles. (D) Taxol loading and release by two different methods fitted with two first order functions for a burst release phase (E) 1st phase and a second, sustained release phase (F).

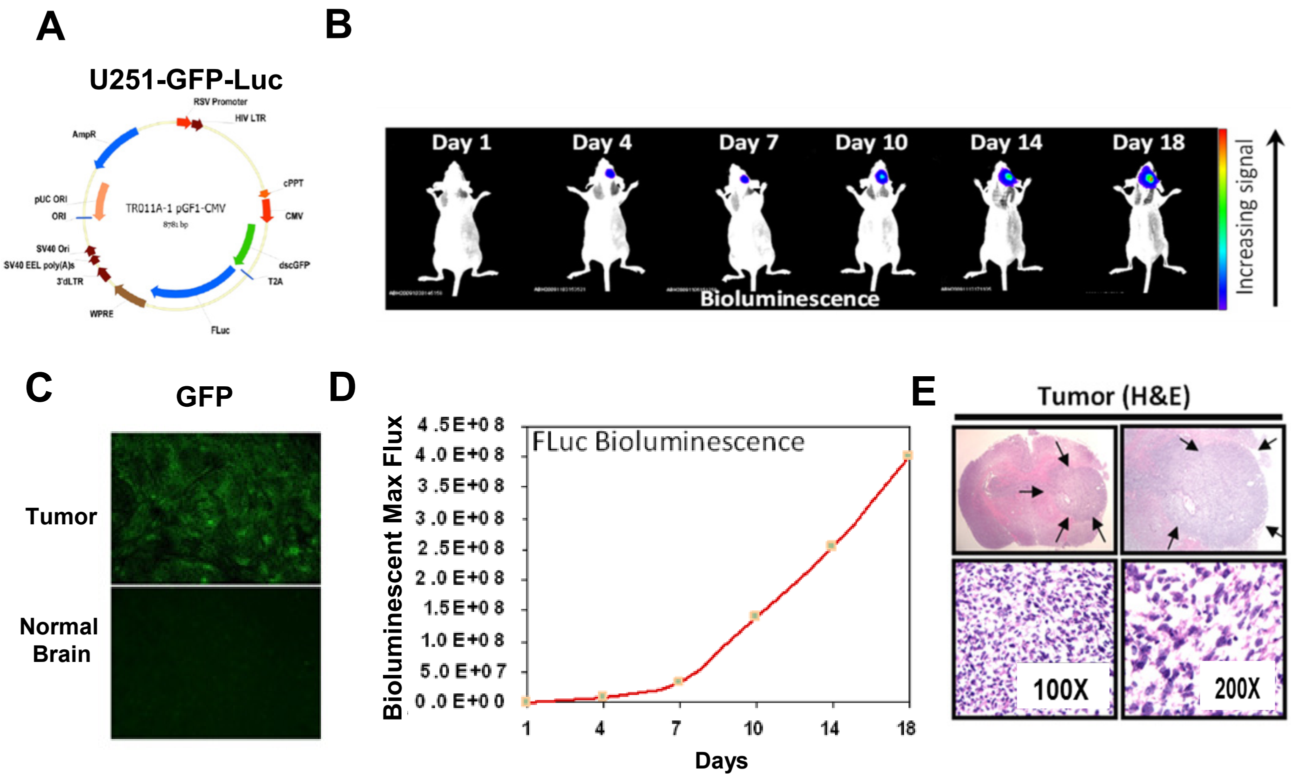

Supplemental Figure 2: Establishing bio-imageable tumors from human GBM cells. (A) Engineered lentivector plasmid encoding for green fluorescent protein (GFP) and luciferase was stably integrated into U251 human-derived GBM cells. (B) Bioluminescent imaging (BLI) shows serial growth of the implanted tumors (representative mice shown during BLI on the specified day after initial implantation of tumor cells). (C) Fluorescent microscopy (GFP) distinguishes GFP-expressing tumor cells (top) from normal brain tissue. (D) Bioluminescence flux reflects the rapid intracranial growth patterns associated with GBM. U251-GFP-Luc cells were stereotactically implanted, followed by in vivo serial BLI of the resultant brain tumors. BLI flux was defined as photons per second/cm2. (E) Hematoxylin/eosin staining of brains excised from mice shows the orthotopic tumor, designated with arrows, under light microscopy at four different magnifications.

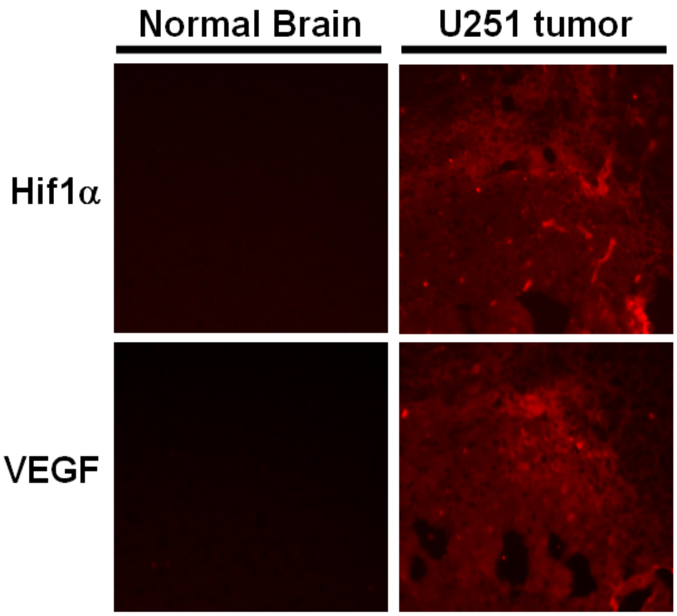

Supplemental Figure 3: Orthotopic GBM tumors show robust expression of Hif1-α and VEGF. Normal brain (left column) or orthotopic U251 brain tumor tissue (right column) were sectioned, fixed onto slides, and stained for hypoxia-inducible factor-alpha (Hif1-α) (top) or VEGF (bottom) protein expression. The immunofluorescent analyses were performed via the respective specific primary antibodies followed red fluorescent protein (RFP)-labeled secondary antibodies to detect the specific signal.

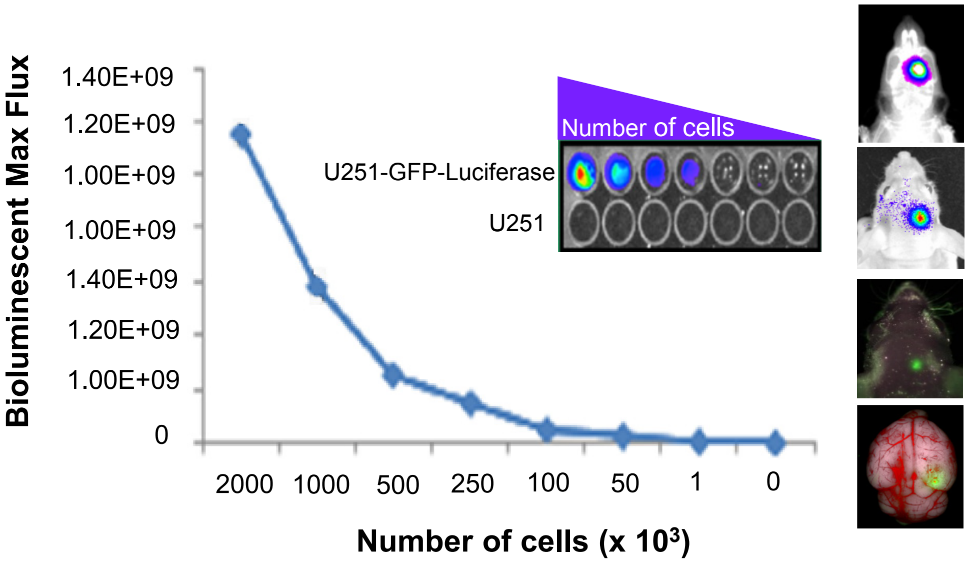

Supplemental Figure 4: Bioluminescent image (BLI) signal correlates with the number of GBM cells in culture. Graph showing the Bioluminescence flux associated with specific numbers of human GBM (U251-GFP-Luc) cells exposed to D-luciferin. Right-hand panels show representative images of signal evident when the BLI-capable GBM cells are implanted orthotopically into the brains of mice. As an additional confirmation of tumor growth, the GFP fluorescence is also detectable when viewed under fluorescent conditions, in both the mouse, and in the excised brain after sacrifice.

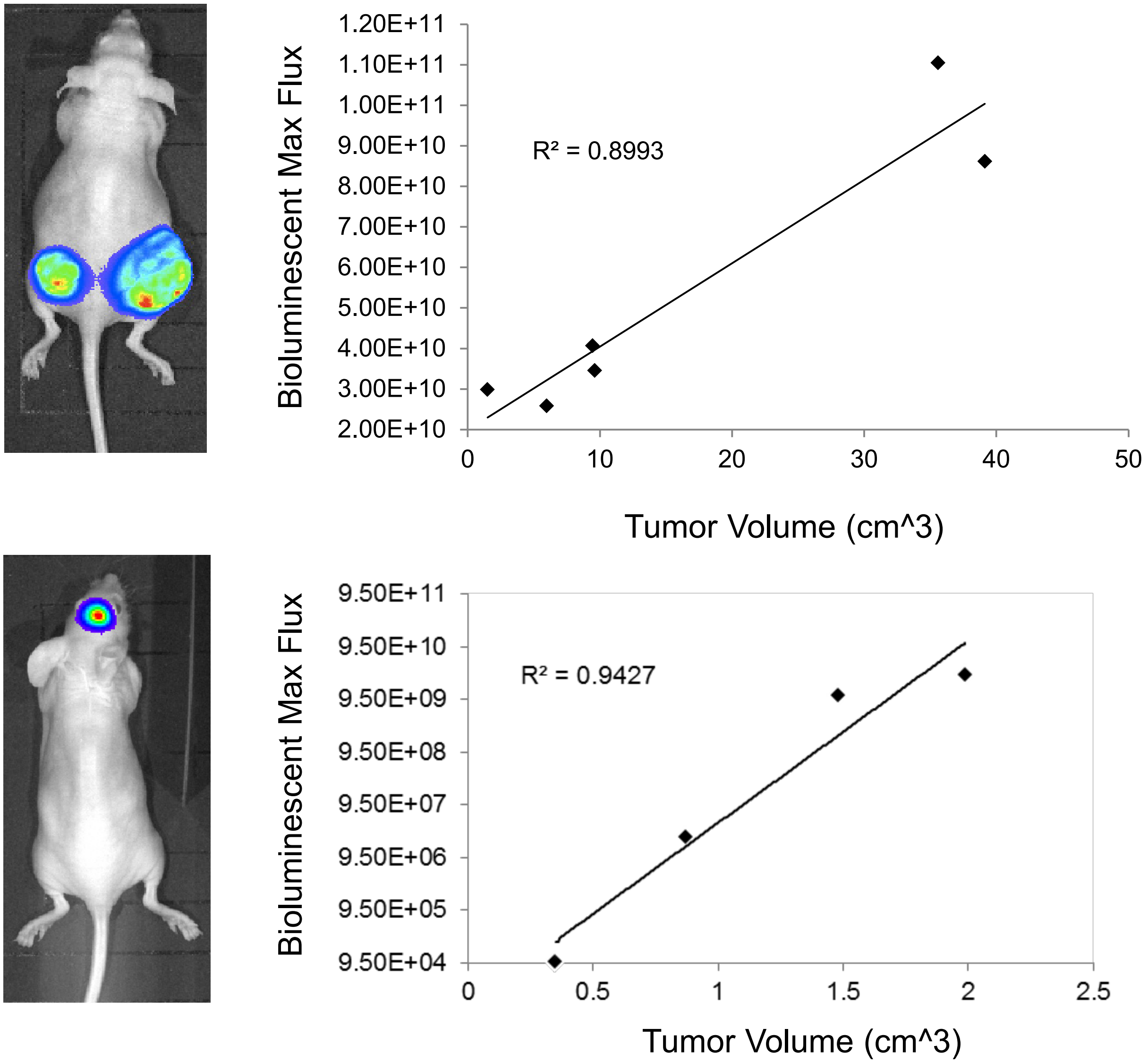

Supplemental Figure 5: Bioluminescent imaging signal from tumors growing within the mouse correlate well with tumor volume measured after excision. In vivo BLI signal ("Bioluminescent Max Flux") was recorded for mice with heterotopic flank tumors (Top) or orthotopic brain tumors (Bottom) immediately prior to sacrifice. The tumors of each mouse were then excised and the respective volume of each tumor was manually measured. The measured tumor volumes significantly correlate with BLI values (R2=0.899).

1	MW	Low %	Mid %	High %	Protein Name (Gene)	Mean R^2	<PRF> / Total	Tot IC IonCurr	Med IC IonCurr	Log(Med IC IonCurr)	Log(IC / SC)	Log(in vitro / SC)	% (IC / SC)	% (in vitro / SC)
2	H				Collagen alpha-1(XIV) chain (COL14A1)	0.939	7.0 / 19	5.0E+07	1.5E+06	6.2	1.19	-0.67	16 ± 1	0 ± 0
3	M				Transforming growth factor, beta-induced, 68kDa variant (TGFBI)	0.870	3.0 / 4	4.9E+07	9.7E+06	7.0	1.38	1.26	24 ± 1	18 ± 1
4	H				Tenascin (TNC)	0.905	13.0 / 23	1.0E+08	2.8E+06	6.5	1.53	0.55	34 ± 1	4 ± 0
5	L				Glial fibrillary acidic protein (GFAP)	0.919	4.5 / 9	3.5E+07	1.9E+06	6.3	1.68	-0.43	48 ± 5	0 ± 0
6	M				4F2 cell-surface antigen heavy chain (SLC3A2)	0.949	5.0 / 8	6.3E+08	8.5E+07	7.9	1.69	2.63	49 ± 3	429 ± 31
7	L				Creatine kinase B-type (CKB)	0.535	3.0 / 3	2.8E+08	1.0E+08	8.0	1.72	1.73	53 ± 5	53 ± 9
8	L				Eukaryotic translation initiation factor 4B (EIF4B)	0.806	3.0 / 3	3.4E+07	7.1E+06	6.8	1.73	2.35	54 ± 5	225 ± 37
9	H				Collagen alpha-1(XVIII) chain (COL18A1)	0.947	3.0 / 3	2.8E+07	6.5E+06	6.8	1.75	1.22	57 ± 2	17 ± 1
10	M				Periostin (POSTN)	0.919	3.5 / 10	3.8E+07	8.4E+05	5.9	1.76	0.11	58 ± 3	1 ± 0
11	L				Heterogeneous nuclear ribonucleoproteins C1/C2 (HNRNPC)	0.920	3.0 / 3	1.7E+08	3.6E+07	7.6	1.77	1.69	59 ± 6	49 ± 8
12	L				Fructose-bisphosphate aldolase A (ALDOA)	0.955	3.0 / 4	1.3E+09	2.4E+08	8.4	1.79	1.81	61 ± 6	65 ± 10
13	M				Galectin-3-binding protein (LGALS3BP)	0.953	3.5 / 4	7.2E+07	1.8E+07	7.2	1.80	1.51	63 ± 4	32 ± 2
14	M		4.4		Glycogen phosphorylase, brain form (PYGB)	0.974	4.0 / 4	2.4E+08	6.6E+07	7.8	1.82	1.89	65 ± 4	77 ± 6
15	L				Vimentin (VIM)	0.913	5.5 / 6	8.3E+09	1.5E+09	9.2	1.83	1.54	68 ± 7	35 ± 6
16	M				Staphylococcal nuclease domain-containing protein 1 (SND1)	0.777	3.0 / 5	1.5E+08	2.3E+07	7.4	1.83	1.94	68 ± 4	87 ± 6
17	M				Pyruvate kinase isozymes M1/M2 (PKM2)	0.979	5.0 / 6	1.0E+10	1.6E+09	9.2	1.84	1.97	70 ± 4	94 ± 7
18	M				Glucose-6-phosphate isomerase (GPI)	0.934	6.0 / 7	1.1E+09	1.1E+08	8.0	1.84	1.77	70 ± 4	59 ± 4
19	L				60S ribosomal protein L18 (RPL18)	0.990	3.0 / 3	4.7E+08	2.0E+08	8.3	1.84	2.19	70 ± 7	156 ± 25
20	M				Lamin A/C overlap (LMNA)	ND	2.0 / 2	2.9E+08	1.5E+08	8.2	1.85	1.63	71 ± 4	43 ± 3
21	L	25.3			Annexin A1 (ANXA1)	0.974	13.0 / 13	4.4E+09	2.0E+08	8.3	1.85	1.86	71 ± 5	73 ± 11
22	L				Phosphoglycerate kinase 1 (PGK1)	0.976	6.0 / 6	3.8E+09	5.8E+08	8.8	1.86	1.63	72 ± 7	43 ± 7
23	M				Calpain-2 catalytic subunit (CAPN2)	0.620	3.0 / 5	1.6E+08	3.2E+07	7.5	1.86	2.23	72 ± 4	169 ± 12
24	L				Rab GDP dissociation inhibitor beta (GDI2)	0.938	6.0 / 6	4.4E+08	5.8E+07	7.8	1.86	2.06	72 ± 7	116 ± 19
25	H				Non-muscle myosin heavy chain IIa (MYH9)	0.911	21.0 / 30	4.0E+09	6.2E+07	7.8	1.87	2.82	73 ± 3	654 ± 26
26	M				Gelsolin (GSN)	0.909	10.0 / 12	2.1E+08	1.3E+07	7.1	1.87	0.92	73 ± 4	8 ± 1
27	M				T-complex protein 1 subunit zeta (CCTA6A)	0.788	3.0 / 4	9.4E+08	1.5E+08	8.2	1.87	2.70	74 ± 4	496 ± 36
28	L				60S ribosomal protein L14 (RPL14)	0.501	3.0 / 3	1.7E+08	6.6E+07	7.8	1.87	2.16	75 ± 7	144 ± 23
29	M				6-phosphofructokinase type C (PFKP)	0.947	6.5 / 8	4.5E+08	5.6E+07	7.8	1.88	1.98	76 ± 4	97 ± 7
30	L				BRCA2 and CDKN1A-interacting protein (BCCIP)	0.961	3.0 / 3	1.7E+08	3.6E+07	7.6	1.89	2.20	78 ± 8	159 ± 26
31	L				Peroxiredoxin-1 (PRDX1)	0.966	3.0 / 3	3.2E+08	1.0E+08	8.0	1.90	1.75	79 ± 8	57 ± 9
32	M				Phenylalanyl-tRNA synthetase beta chain (FARSB)	0.930	3.5 / 4	1.2E+08	2.2E+07	7.3	1.90	2.28	79 ± 5	190 ± 14
33	M				Delta-1-pyrroline-5-carboxylate synthase (ALDH18A1)	0.981	3.0 / 3	8.7E+07	2.9E+07	7.5	1.90	2.29	79 ± 5	195 ± 14
34	L				Coactosin-like protein (COTL1)	0.728	3.0 / 3	3.6E+08	1.3E+08	8.1	1.90	2.15	80 ± 8	142 ± 23
35	L				Alpha-enolase (ENO1)	0.947	9.0 / 9	7.1E+09	7.2E+08	8.9	1.90	1.75	80 ± 8	56 ± 9
36	L				Elongation factor 1-delta (EEF1D)	0.927	4.5 / 5	1.5E+09	2.8E+08	8.4	1.90	2.38	80 ± 8	238 ± 39
37	H			5.7	Bifunctional aminoacyl-tRNA synthetase (EPRS)	0.920	10.5 / 16	4.2E+08	2.4E+07	7.4	1.90	1.85	80 ± 3	70 ± 3
38	L				L-lactate dehydrogenase B chain (LDHB)	0.958	4.0 / 4	2.1E+09	5.0E+08	8.7	1.91	2.23	80 ± 8	170 ± 28
39	L				Superoxide dismutase [Cu-Zn] (SOD1)	0.880	3.0 / 3	2.4E+08	9.7E+07	8.0	1.91	1.70	81 ± 8	50 ± 8
40	L				ATP synthase subunit b, mitochondrial (ATP5F1)	0.774	3.0 / 3	9.9E+07	4.3E+07	7.6	1.91	2.13	81 ± 8	136 ± 22
41	H				Filamin-B (FLNB)	0.913	32.0 / 36	8.9E+08	2.1E+07	7.3	1.91	1.54	81 ± 3	35 ± 1
42	L				60S ribosomal protein L6 (RPL6)	0.955	3.0 / 4	4.4E+08	1.0E+08	8.0	1.91	2.24	81 ± 8	174 ± 28
43	M				Catalase (CAT)	0.924	3.0 / 3	5.6E+07	1.9E+07	7.3	1.91	1.73	82 ± 5	53 ± 4
44	M				Phosphoglucomutase-1 (PGM1)	0.941	4.5 / 5	3.7E+07	7.1E+06	6.8	1.92	1.14	83 ± 5	14 ± 1
45	L				Thioredoxin domain-containing protein 5 (TXNDC5)	0.839	3.0 / 6	2.0E+08	2.8E+07	7.4	1.92	2.09	83 ± 8	123 ± 20
46	M				Arginyl-tRNA synthetase, cytoplasmic (RARS)	0.822	3.0 / 4	2.6E+08	6.7E+07	7.8	1.92	2.45	83 ± 5	281 ± 20
47	M				T-complex protein 1 subunit theta (CCT8)	0.904	4.5 / 7	7.6E+08	8.9E+07	8.0	1.92	2.37	83 ± 5	234 ± 17
48	H			3.2	Eukaryotic translation initiation factor 4 gamma 1 (EIF4G1)	0.912	8.0 / 11	3.0E+08	2.2E+07	7.3	1.92	2.02	84 ± 4	104 ± 5
49	M				Moesin (MSN)	0.991	3.0 / 4	6.7E+08	1.4E+08	8.1	1.92	2.00	84 ± 5	99 ± 7
50	L				F-actin-capping protein subunit alpha-1 (CAPZA1)	0.932	3.0 / 3	1.1E+08	2.3E+07	7.4	1.93	1.83	85 ± 8	68 ± 11
51	M				Protein disulfide-isomerase (P4HB)	0.909	11.0 / 11	2.1E+09	1.0E+08	8.0	1.93	1.92	85 ± 5	83 ± 6
52	H				Ras GTPase-activating-like protein (IQGAP1)	0.923	19.5 / 20	8.7E+08	3.3E+07	7.5	1.93	1.71	85 ± 4	51 ± 2
53	L				Nuclear migration protein (NUDC)	0.949	3.5 / 4	1.9E+08	3.9E+07	7.6	1.93	2.31	86 ± 8	205 ± 33
54	M		8.5		Nucleolin (NCL)	0.909	9.0 / 10	2.3E+09	1.8E+08	8.2	1.93	2.16	86 ± 5	144 ± 13
55	M				Alpha-actinin-4 (ACTN4)	0.930	5.0 / 5	2.7E+08	5.0E+07	7.7	1.94	1.43	87 ± 5	27 ± 2
56	L				Ubiquitin carboxyl-terminal hydrolase isozyme L1 (UCHL1)	0.727	3.0 / 3	1.4E+09	4.5E+08	8.7	1.94	1.87	87 ± 9	75 ± 12
57	M				Interleukin enhancer-binding factor 3 (ILF3)	0.964	3.0 / 3	1.7E+08	8.0E+07	7.9	1.94	1.87	87 ± 5	75 ± 5
58	L				Fatty acid-binding protein, brain (FABP7)	0.983	4.5 / 5	1.1E+08	1.1E+07	7.0	1.94	0.56	87 ± 9	4 ± 1
59	L				Cytochrome b-c1 complex subunit 2, mitochondrial (UQCRC2)	0.982	3.5 / 5	2.4E+08	4.5E+07	7.7	1.94	2.11	88 ± 9	129 ± 21
60	L				Cathepsin D (CTSD)	0.934	7.0 / 7	1.3E+08	2.0E+07	7.3	1.94	1.11	88 ± 9	13 ± 2
61	L				Hyaluronan-binding protein 1 (C1QBP)	0.756	3.0 / 5	1.6E+09	3.8E+08	8.6	1.94	2.68	88 ± 9	474 ± 77
62	M				Cytoskeleton-associated protein 4 (CKAP4)	0.929	9.5 / 11	6.8E+08	4.5E+07	7.7	1.95	2.06	88 ± 5	116 ± 8
63	L	14.1			Heat shock protein beta-1 (HSPB1)	0.973	5.0 / 6	9.2E+08	1.6E+08	8.2	1.95	1.97	88 ± 9	93 ± 16
64	H				Talin-1 (TLN)	0.945	15.0 / 15	6.0E+08	3.3E+07	7.5	1.95	1.69	88 ± 4	50 ± 2
65	L				Protein DJ-1 (PARK7)	0.970	4.0 / 5	2.8E+08	5.3E+07	7.7	1.95	1.75	89 ± 9	56 ± 9
66	M				Bifunctional purine biosynthesis protein PURH (ATIC)	0.938	12.5 / 13	9.5E+08	5.2E+07	7.7	1.95	2.31	90 ± 5	204 ± 15
67	M		6.1		CD44 antigen (CD44)	0.937	5.0 / 6	7.0E+08	1.0E+08	8.0	1.95	1.87	90 ± 5	74 ± 5
68	M				Nucleolar RNA helicase 2 (DDX21)	0.942	3.5 / 7	3.9E+08	6.9E+07	7.8	1.95	2.62	90 ± 5	415 ± 30
69	M				Zyxin (ZYX)	0.990	3.0 / 3	8.5E+07	1.5E+07	7.2	1.95	1.86	90 ± 5	72 ± 5
70	H			33.2	Plectin (isoforms 1, 2, 3, 4, 5, 6, 7, 8 & 9) (PLEC)	0.915	78.0 / 79	2.6E+09	2.3E+07	7.4	1.96	2.01	91 ± 4	103 ± 6
71	M				Dihydropyrimidinase-related protein 2 (DPYSL2)	0.901	4.0 / 5	1.8E+08	4.7E+07	7.7	1.96	1.73	91 ± 5	53 ± 4
72	H			3.5	Nucleoprotein (TPR)	0.923	8.5 / 10	2.7E+08	1.6E+07	7.2	1.96	1.95	91 ± 4	88 ± 5
73	H			1.3	Eukaryotic translation initiation factor 3, subunit A (EIF3A)	0.766	3.0 / 3	2.9E+08	1.2E+08	8.1	1.96	2.06	91 ± 5	114 ± 5
74	M		19.3		X-ray repair cross-complementing protein 5 (XRCC5)	0.908	16.0 / 18	1.9E+09	8.4E+07	7.9	1.96	2.01	92 ± 5	102 ± 9
75	H				UDP-glucose:glycoprotein glucosyltransferase 1 (UGGT1)	0.924	3.5 / 4	9.8E+07	1.8E+07	7.3	1.96	1.77	92 ± 4	58 ± 2
76	M				78 kDa gastrin-binding protein (HADHA)	0.859	5.5 / 8	3.9E+08	3.7E+07	7.6	1.97	2.12	93 ± 5	131 ± 9
77	M				Lysyl-tRNA synthetase (KARS)	0.968	5.0 / 5	4.5E+08	6.2E+07	7.8	1.97	2.53	93 ± 5	342 ± 25
78	M				60 kDa heat shock protein, mitochondrial (HSPD1)	0.984	6.5 / 8	7.5E+09	7.4E+08	8.9	1.97	2.68	93 ± 5	480 ± 34
79	M				Importin subunit alpha-2 (KPNA2)	0.960	4.5 / 6	5.6E+08	5.0E+07	7.7	1.97	2.77	94 ± 5	593 ± 43
80	M				Ezrin (EZR)	0.945	3.5 / 4	3.5E+08	7.4E+07	7.9	1.97	2.26	94 ± 5	183 ± 13
81	M				T-complex protein 1 subunit epsilon (CCT5)	0.923	4.5 / 8	9.1E+08	7.8E+07	7.9	1.97	2.57	94 ± 5	374 ± 27
82	H			1.9	Translational activator GCN1 (GCN1L1)	0.864	3.0 / 6	7.2E+07	1.3E+07	7.1	1.97	2.29	94 ± 4	196 ± 15
83	M				Replication protein A 70 kDa DNA-binding subunit (RPA1)	0.926	3.5 / 4	4.5E+07	1.1E+07	7.0	1.98	1.77	95 ± 5	58 ± 4
84	M				GMP synthase [glutamine-hydrolyzing] (GMPS)	0.934	3.0 / 3	8.1E+07	3.1E+07	7.5	1.98	2.24	95 ± 5	172 ± 12
85	M				Alanyl-tRNA synthetase, cytoplasmic (AARS)	0.687	3.0 / 3	8.7E+07	1.7E+07	7.2	1.98	2.06	95 ± 6	115 ± 8
86	H				Spectrin beta chain, brain 1 (SPTBN1)	0.927	7.5 / 8	1.6E+08	1.6E+07	7.2	1.98	1.61	95 ± 4	41 ± 2
87	L				Triosephosphate isomerase (TPI1)	0.961	3.0 / 4	6.0E+08	1.4E+08	8.1	1.98	1.84	95 ± 9	69 ± 11
88	M				Stress-induced-phosphoprotein 1 (STIP1)	0.694	3.0 / 3	2.4E+08	5.0E+07	7.7	1.98	2.45	96 ± 6	279 ± 20
89	H			1.7	Cytoskeleton-associated protein 5 (CKAP5)	0.971	4.0 / 4	7.4E+07	1.1E+07	7.0	1.98	2.06	96 ± 4	116 ± 5
90	L				Annexin A2 (ANXA2)	0.965	5.0 / 5	1.6E+09	3.1E+08	8.5	1.99	2.34	97 ± 10	219 ± 36
91	M				Protein disulfide-isomerase A3 (PDIA3)	0.949	10.0 / 10	6.2E+09	5.3E+08	8.7	1.99	1.98	97 ± 6	95 ± 7
92	M				Polypyrimidine tract-binding protein 1 (PTBP1)	0.933	3.5 / 5	6.8E+08	1.1E+08	8.0	1.99	2.14	97 ± 6	138 ± 10
93	M				tRNA (cytosine(34)-C(5))-methyltransferase (NSUN2)	0.717	3.0 / 3	1.2E+08	4.7E+07	7.7	1.99	2.47	98 ± 6	294 ± 21
94	M				Transketolase (TKT)	0.981	10.0 / 10	4.5E+09	2.4E+08	8.4	1.99	2.21	98 ± 6	163 ± 12
95	M				Heat shock 70 kDa protein 4 (HSPA4)	0.881	3.0 / 3	3.2E+08	8.6E+07	7.9	2.00	2.32	99 ± 6	211 ± 15
96	H				Filamin A (FLNA)	0.925	27.5 / 29	4.3E+09	1.0E+08	8.0	2.00	1.64	99 ± 4	44 ± 2
97	H				Microtubule-associated protein 1B (MAP 1B)	0.911	11.5 / 16	4.5E+08	2.6E+07	7.4	2.00	2.12	99 ± 4	132 ± 5
98	M				Thioredoxin reductase 1, cytoplasmic (TXNRD1)	0.761	3.0 / 6	4.2E+08	5.9E+07	7.8	2.00	2.95	100 ± 6	900 ± 65
99	M				Elongation factor 2 (EEF2)	0.977	3.5 / 4	1.6E+09	4.2E+08	8.6	2.00	2.31	100 ± 6	205 ± 15
100	L				Annexin A5 (ANXA5)	0.963	3.5 / 4	9.1E+08	2.7E+08	8.4	2.00	1.75	101 ± 10	56 ± 9

	function
Collagen alpha-1(XIV) chain (COL14A1)	*one of several non-fibril, non-basement membrane, Fibril Associated Collagens with Interrupted Triple helices*
Transforming growth factor, beta-induced, 68kDa variant (TGFBI)	*RGD-containing protein that binds collagens I, II and IV . The protein is induced by transforming growth factor-beta and acts to inhibit cell adhesion*
Tenascin (TNC)	*interacts with fibronectin; invasive edges of GBM and associated with vascular sprouting*
Glial fibrillary acidic protein (GFAP)	*maintain mechanical strength as well as the shape of cells; marker of neurologic damage after stroke and brain injury*
4F2 cell-surface antigen heavy chain (SLC3A2)	*associates with integrins and mediates integrin-dependent signaling related to normal cell growth and tumorigenesis*

101	L	10.9			Peroxiredoxin-6 (PRDX6)	0.937	4.5 / 5	4.1E+08	5.8E+07	7.8	2.01	2.10	102 ± 11	127 ± 21
102	M				Dihydropyrimidinase-related protein 3 (DPYSL3)	0.954	5.0 / 7	5.1E+08	7.8E+07	7.9	2.01	1.45	102 ± 6	28 ± 2
103	M				ATP-citrate synthase (ACLY)	0.969	3.0 / 3	1.9E+08	3.3E+07	7.5	2.01	2.19	102 ± 6	156 ± 11
104	L				Stomatin-like protein 2 (STOML2)	0.837	3.5 / 4	3.0E+08	7.3E+07	7.9	2.01	2.55	103 ± 10	353 ± 57
105	M				DNA replication licensing factor (MCM6)	0.585	3.0 / 3	6.7E+07	2.1E+07	7.3	2.02	2.04	104 ± 6	110 ± 8
106	M				Ubiquitin-like modifier-activating enzyme 1 (UBA1)	0.932	3.5 / 6	5.1E+08	8.2E+07	7.9	2.02	2.11	104 ± 6	127 ± 9
107	L	6.6			S-methyl-5'-thioadenosine phosphorylase (MTAP)	0.965	3.0 / 3	2.4E+08	7.2E+07	7.9	2.02	2.09	104 ± 11	124 ± 19
108	H				Spectrin alpha chain, brain (SPTAN1)	0.953	10.5 / 11	1.6E+08	7.7E+06	6.9	2.02	1.73	104 ± 4	54 ± 2
109	M				Very long-chain specific acyl-CoA dehydrogenase, mitochondrial (ACA	0.942	6.0 / 6	2.3E+08	4.3E+07	7.6	2.02	2.06	104 ± 6	114 ± 8
110	M				Heat shock protein 105 kDa (HSPH1)	0.781	3.0 / 5	3.5E+08	8.8E+07	7.9	2.02	2.77	105 ± 6	593 ± 43
111	M				T-complex protein 1 subunit beta (CCT2)	0.920	6.0 / 8	6.7E+08	6.5E+07	7.8	2.02	2.55	105 ± 6	353 ± 25
112	H			2.0	Nuclear pore complex protein (NUP160)	0.563	3.0 / 6	8.1E+07	1.3E+07	7.1	2.02	2.19	106 ± 5	155 ± 10
113	M				Ribophorin-1 (RPN1)	0.954	6.5 / 7	5.7E+08	5.6E+07	7.8	2.02	2.09	106 ± 6	123 ± 9
114	M		5.4		Far upstream element-binding protein 2 (KHSRP)	0.926	5.0 / 5	3.0E+08	6.0E+07	7.8	2.03	2.05	106 ± 6	113 ± 9
115	L				Carbonyl reductase [NADPH] 1 (CBR1)	0.995	3.0 / 4	3.1E+08	5.5E+07	7.7	2.03	1.76	106 ± 10	58 ± 9
116	H				CAD Protein (CAD)	0.948	5.5 / 8	2.0E+08	2.0E+07	7.3	2.03	2.27	107 ± 4	185 ± 7
117	L				Galectin-1 (LGALS1)	0.604	3.0 / 5	3.8E+08	7.4E+07	7.9	2.03	1.22	107 ± 11	17 ± 3
118	H				Fatty acid synthase (FASN)	0.903	63.5 / 71	9.1E+09	7.6E+07	7.9	2.03	2.56	108 ± 5	366 ± 15
119	M		17.4		X-ray repair cross-complementing protein 6 (XRCC6)	0.930	14.0 / 17	1.3E+09	6.1E+07	7.8	2.04	2.01	109 ± 7	101 ± 9
120	H				A-kinase anchor protein 12 (AKAP12)	0.919	37.5 / 41	3.1E+09	4.2E+07	7.6	2.04	2.62	109 ± 5	415 ± 17
121	M		11.6		Lamin-B2 (LMNB2)	0.937	7.0 / 10	3.8E+08	3.7E+07	7.6	2.04	2.03	109 ± 7	106 ± 9
122	M		10.9		C-1-tetrahydrofolate synthase, cytoplasmic (MTHFD1)	0.950	8.5 / 9	3.5E+08	3.5E+07	7.5	2.04	2.12	109 ± 7	131 ± 9
123	M				Heat shock protein 75 kDa, mitochondrial (TRAP1)	0.964	3.0 / 4	1.6E+08	4.2E+07	7.6	2.04	2.62	110 ± 6	418 ± 30
124	M				Threonyl-tRNA synthetase, cytoplasmic (TARS)	0.970	4.0 / 6	2.4E+08	4.1E+07	7.6	2.04	2.32	110 ± 6	208 ± 15
125	L	5.6			Inorganic pyrophosphatase (PPA1)	0.891	3.0 / 3	2.7E+08	7.8E+07	7.9	2.04	2.21	111 ± 11	161 ± 26
126	M				Glucose-6-phosphate 1-dehydrogenase (G6PD)	0.917	5.0 / 6	2.5E+08	3.0E+07	7.5	2.05	2.37	111 ± 6	232 ± 17
127	L	7.7			Peroxiredoxin-2 (PRDX2)	0.774	3.0 / 4	3.6E+08	9.4E+07	8.0	2.05	1.93	111 ± 11	85 ± 14
128	H			4.3	Cytoplasmic dynein 1 heavy chain 1 (DYNC1H1)	0.932	9.5 / 11	4.3E+08	2.5E+07	7.4	2.05	1.99	112 ± 5	98 ± 5
129	H				DNA (cytosine-5)-methyltransferase 1 (DNMT1)	0.938	4.0 / 4	6.5E+07	1.6E+07	7.2	2.05	2.44	113 ± 5	274 ± 11
130	H				Neuroblast differentiation-associated protein (AHNAK)	0.936	7.0 / 11	2.7E+08	1.5E+07	7.2	2.06	2.34	114 ± 5	221 ± 9
131	L	7.4			Protein disulfide-isomerase A6 (PDIA6)	0.940	4.0 / 4	3.6E+08	6.3E+07	7.8	2.06	1.93	114 ± 13	86 ± 14
132	L				Tropomyosin alpha-4 chain (TPM4)	0.905	3.5 / 4	1.8E+08	4.7E+07	7.7	2.06	1.85	114 ± 11	70 ± 11
133	L				Phosphatidylethanolamine-binding protein 1 (PEBP1)	0.921	4.0 / 5	6.1E+08	8.6E+07	7.9	2.06	1.90	115 ± 11	79 ± 13
134	L				Glyceraldehyde-3-phosphate dehydrogenase (GAPDH)	0.977	6.5 / 7	1.0E+10	1.3E+09	9.1	2.06	1.89	116 ± 11	78 ± 13
135	L				Estradiol 17-beta-dehydrogenase 12 (HSD17B12)	0.882	3.0 / 3	1.5E+08	4.9E+07	7.7	2.06	2.10	116 ± 11	126 ± 21
136	H			43.2	DNA-dependent protein kinase catalytic subunit (PRKDC)	0.910	97.0 / 101	4.5E+09	3.0E+07	7.5	2.06	1.99	116 ± 5	97 ± 6
137	M				Lamina-associated polypeptide 2, isoform alpha (TMPO)	ND	2.0 / 2	1.3E+08	6.3E+07	7.8	2.07	2.11	117 ± 7	128 ± 9
138	M		13.0		Calnexin (CANX)	0.934	12.5 / 14	2.7E+09	1.2E+08	8.1	2.07	2.00	117 ± 8	100 ± 7
139	H				Tumor suppressor p53-binding protein 1 (TP53BP1)	0.952	4.0 / 6	3.9E+07	5.6E+06	6.7	2.07	1.83	117 ± 5	68 ± 3
140	H				Nestin (NES)	0.905	37.5 / 51	2.4E+09	4.4E+07	7.6	2.07	1.68	117 ± 5	48 ± 2
141	M				Transcription intermediary factor 1-beta (TRIM28)	0.905	3.0 / 4	1.8E+08	3.2E+07	7.5	2.07	2.02	117 ± 7	105 ± 8
142	M				Lamin B1 (LMNB1)	0.978	5.0 / 5	9.8E+08	1.0E+08	8.0	2.07	1.95	119 ± 7	89 ± 6
143	M				Protein disulfide-isomerase A4 (PDIA4)	0.951	10.5 / 11	5.1E+08	3.6E+07	7.6	2.08	1.83	119 ± 7	68 ± 5
144	M				Alpha-actinin-1 (ACTN1)	0.798	3.5 / 4	1.6E+08	2.7E+07	7.4	2.08	1.34	120 ± 7	22 ± 2
145	M				Apoptosis-inducing factor 1, mitochondrial (AIFM1)	0.813	3.0 / 3	8.8E+07	2.7E+07	7.4	2.08	2.37	120 ± 7	236 ± 17
146	H				Peroxidasin homolog (PXDN)	0.913	9.5 / 15	5.5E+07	1.9E+06	6.3	2.09	0.81	122 ± 5	6 ± 0
147	M				Eukaryotic translation initiation factor 4B (EIF4B)	0.959	4.0 / 4	1.0E+08	1.7E+07	7.2	2.09	2.30	122 ± 7	200 ± 14
148	H				Nuclear mitotic apparatus protein 1 (NUMA1)	0.907	13.5 / 18	2.4E+08	1.2E+07	7.1	2.09	1.72	123 ± 5	52 ± 2
149	L	9.8			Glutathione S-transferase P (GSTP1)	0.976	5.0 / 6	1.3E+09	7.6E+07	7.9	2.09	2.00	124 ± 12	101 ± 18
150	M				Heterogeneous nuclear ribonucleoprotein U (HNRNPU)	0.979	6.0 / 6	9.0E+08	3.5E+07	7.5	2.10	1.90	125 ± 7	80 ± 6
151	L				6-phosphogluconate dehydrogenase, decarboxylating (PGD)	0.870	3.0 / 5	1.5E+08	3.0E+07	7.5	2.10	1.73	126 ± 12	54 ± 9
152	M				Vinculin (VCL)	0.966	3.5 / 4	1.3E+08	2.8E+07	7.4	2.12	1.82	131 ± 8	67 ± 5
153	M				Neutral alpha-glucosidase AB (GANAB)	0.946	14.0 / 14	1.1E+09	6.4E+07	7.8	2.12	2.09	133 ± 8	122 ± 9
154	M				Heat shock 70 kDa protein 1A/1B (HSPA1A/B)	0.792	3.0 / 5	3.2E+08	5.6E+07	7.7	2.13	2.34	135 ± 8	217 ± 16
155	M				Mannosyl-oligosaccharide glucosidase (MOGS)	0.822	3.0 / 3	4.3E+07	2.0E+07	7.3	2.13	1.76	135 ± 8	58 ± 4
156	M				Caldesmon (CALD1)	0.933	9.5 / 10	1.7E+08	1.5E+07	7.2	2.14	1.49	137 ± 8	31 ± 2
157	M				Endoplasmin (HSP90B1)	0.936	7.5 / 8	1.2E+09	1.1E+08	8.0	2.14	1.80	138 ± 8	64 ± 5
158	M				Calreticulin (CALR)	0.680	3.0 / 3	4.1E+08	1.3E+08	8.1	2.15	2.37	140 ± 8	234 ± 17
159	L				Flap endonuclease 1 (FEN1)	0.791	3.0 / 4	1.8E+08	3.5E+07	7.5	2.18	2.39	151 ± 15	245 ± 40
160	M				Glucosidase 2 subunit beta (PRKCSH)	0.949	5.0 / 8	6.1E+08	7.2E+07	7.9	2.20	2.28	160 ± 9	189 ± 14
161	L				Malate dehydrogenase (MDH2)	0.847	3.0 / 4	2.4E+08	6.3E+07	7.8	2.23	2.32	169 ± 17	207 ± 34
162	H				Myoferlin (MYOF)	0.952	6.5 / 9	2.3E+08	2.6E+07	7.4	2.29	2.67	196 ± 8	465 ± 19
163	H				Synemin (SYNM)	0.867	3.5 / 8	5.6E+06	5.2E+05	5.7	2.30	0.75	197 ± 8	6 ± 0
164					*average*	*0.900*							3.1	39.3

Myoferlin (MYOF) *may be involved in membrane regeneration and repair*
Synemin (SYNM) *intermediate filament (IF) primarily functions to integrate mechanical stress and maintain..*

Supplemental Figure 6: Mass spectrometry-determined proteomes of Intracranial Tumor versus Subcutaneous Tumor versus GBM cells in culture. Proteomic comparison between U251 cells grown in culture and SC and IC xenografts of the same cell line grown in mice. The 162 human-derived proteins with 3 or more detected peptides (except LMNA and TMPO with just two peptides) are ranked by the %-difference between IC and SC (i.e. % IC/SC). Only 5 proteins show more than a two-fold difference (COL14A1, TGFBI, TNC, GFAP, SLC3A2), representing just 3.1% of the detected human proteome. All five are structure-related with noted functions in extracellular matrix, adhesion, or cytoskeleton, and all five are lower in brain than in the subcutaneous site. All five proteins also differ by more than two-fold in vitro, for which 39.3% of the detected human proteome differs by more than two-fold relative to flank tumors. Differences between in vivo sites are thus minimal compared to differences with cultured cells. Green (or light gray) indicates a low ratio (or low signal), red (or dark gray) indicates a high ratio (or high signal), and black indicates no change with 100% corresponding to log10(100) = 2. Following lysis and tryptic digestion, peptides uniquely derived from human proteins were identified and quantified using label-free liquid-chromatography coupled tandem mass spectrometry (LC-MS/MS). Samples were analyzed in three molecular weight bands: "L", up to 55 kDa; "M", 55 – 160 kDa; "H", above 160 kDa. Ion currents were normalized against signal from an experimentally determined set of housekeeping proteins (% contributions to the invariant housekeeping set are noted in columns 2 through 4). Analysis was performed using a Peak Ratio Fingerprinting (PRF) algorithm that identified a subset of reliable peptides on which to base quantification i.e. a correlation between pair-wise ratios with $R2$ greater than 0.90, except in cases where there are a limited number of good peptides, in which case the best three are selected and a lower $R2$ is accepted. For this dataset, the overall mean $R2$ = 0.90.

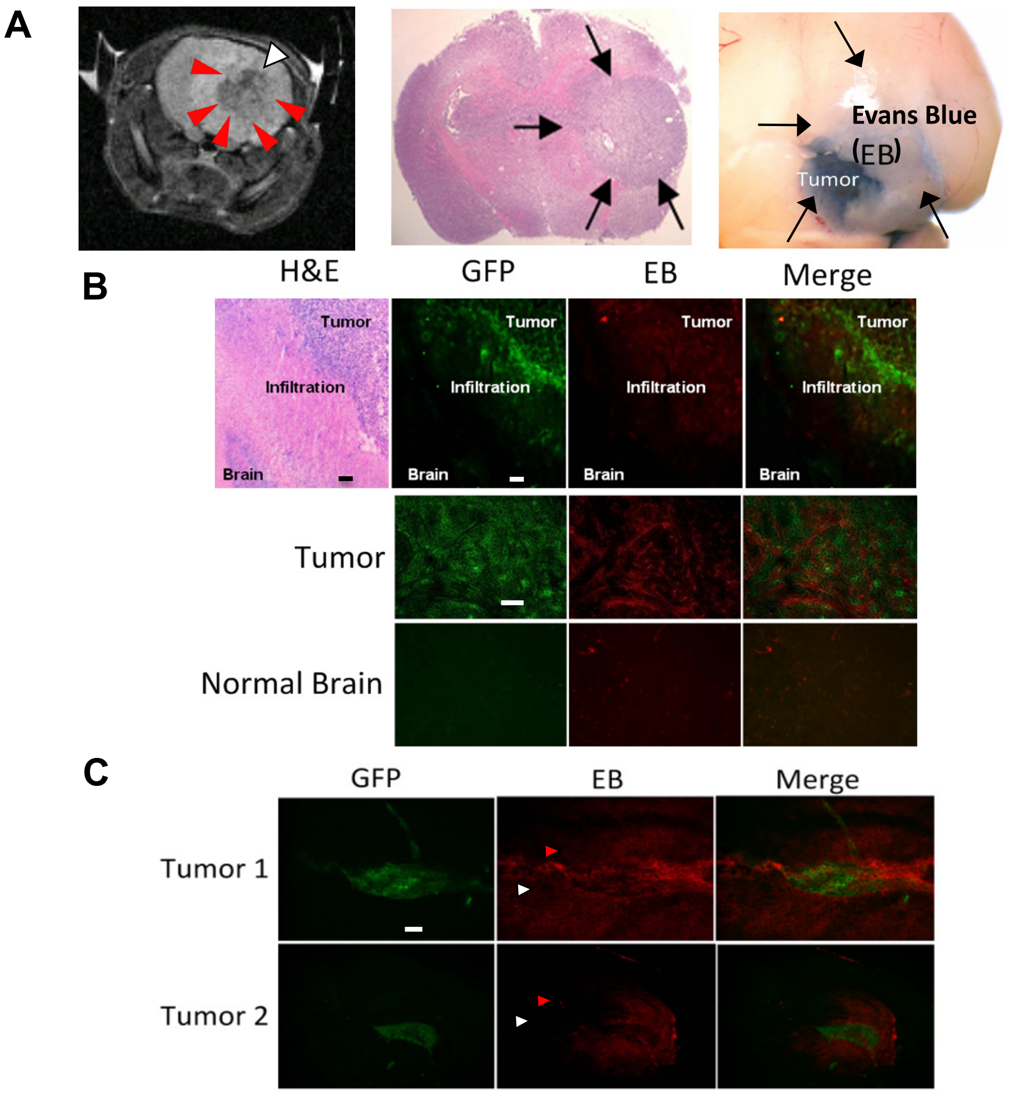

Supplemental Figure 7: Visualization of blood-brain barrier disruption in GBM orthografts via Evans Blue and Immunoglobulin extravasation. (A) Far left: Magnetic resonance imaging (MRI) of a GBM orthograft with red arrows showing the tumor. Middle: Hematoxylin/eosin staining showing infiltration of GBM tumor cells into normal brain tissue. Imaging performed at 2X magnification. Far right: Visualization under light microscopy of an unstained brain removed from a mouse that had been implanted with a GBM tumor orthograft and perfused with Evans Blue (EB) prior to sacrifice. Blue staining due to extravasation of EB within the tumor is clearly visible (denoted by "EB"). Imaging was performed at 10X magnification. (B) Visualization of tumor cells and normal brain tissue under either fluorescent (right three columns) or light microscopy (leftmost column, H&E). The panels in each row are of the same region of the tumor (top rows) or normal brain (bottom row). The columns show respectively imaging of tumor cells via GFP, BBB disruption via extravasation of Evans Blue (EB), or both GFP and EB images merged together (indicating that regions of BBB disruption coincide with regions with tumor cells, but were not seen within normal brain tissue). Imaging performed with 2X magnification (top row) or 10X (bottom 2 rows). (C) Additional images of representative tumors (Tumor 1, Tumor 2) under fluorescence microscopy. The panels in each row are of the same region of each tumor. The columns show respectively imaging of tumor cells via GFP, BBB disruption via extravasation of Evans Blue (EB), or both GFP and EB images merged together, indicating that BBB disruption coincided with regions within the brain that harbor tumor cells but was not entirely uniform.

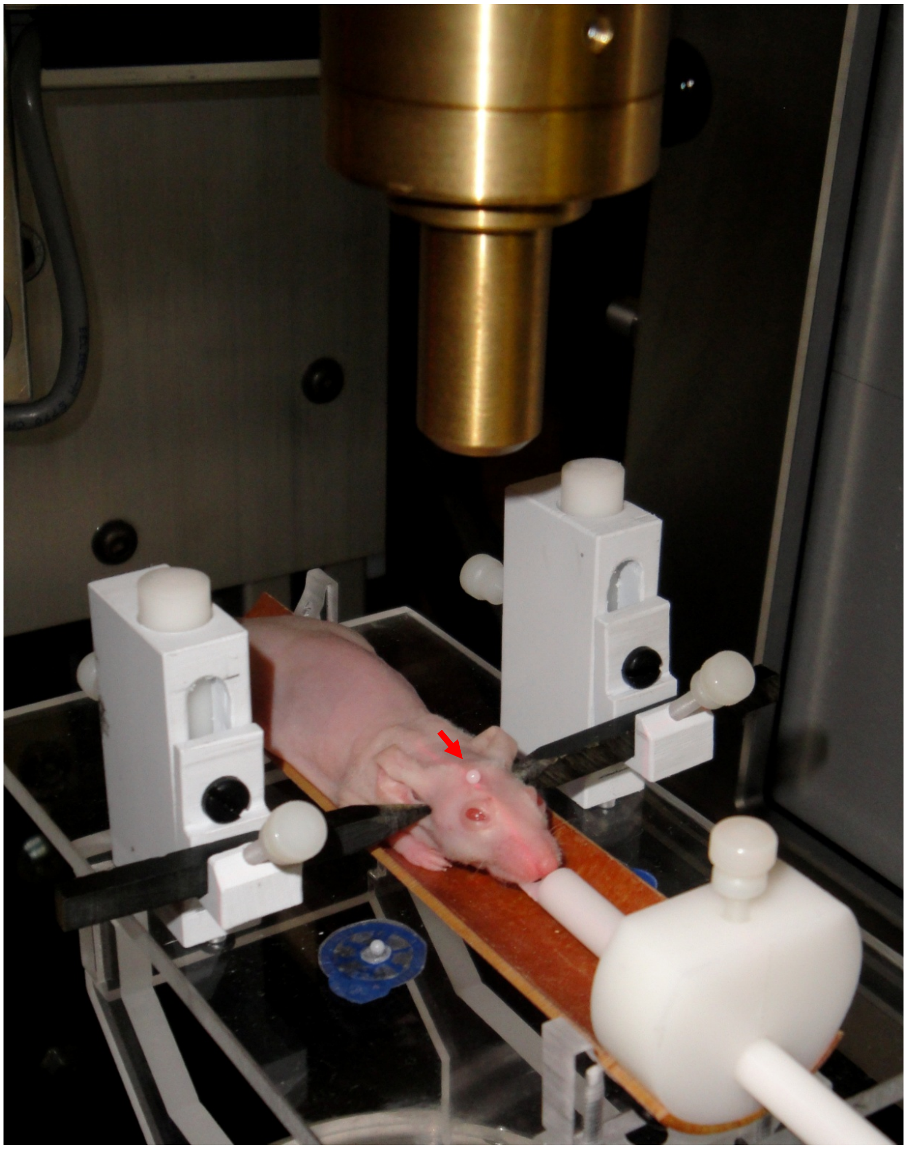

Supplemental Figure 8: Custom-designed stereotactic mouse restrainer for focal brain tumor irradiation using the Small Animal Radiation Research Platform (SARRP). The animal is anchored in place by the bite bar and fully adjustable ear bar system. The arrow indicates a nylon fiducial marker bead placed over the point of maximal BLI signal intensity of the tumor to assist in target delineation using the onboard cone-beam CT scanner. The targeting laser used by the SARRP is faintly visible over the animal's head.

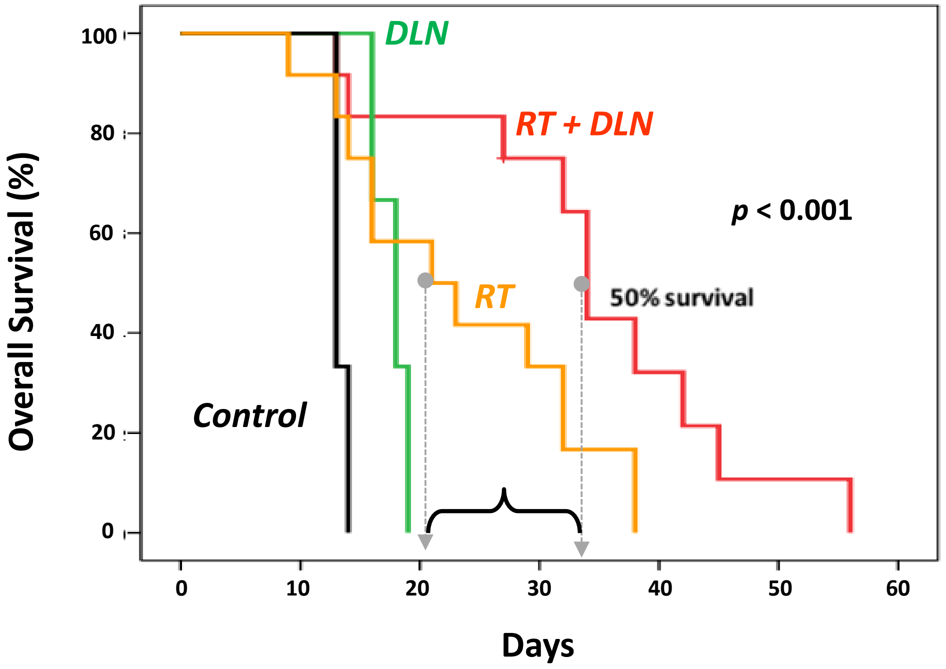

Treatment Group	Median Survival (Days)	Mean Survival (Days)
RT + DLN	34	35
RT	21	23
DLN*	18	18
Control*	13	13

Supplemental Figure 9: Cumulative results of treatment modalities show the best survival is associated with the combination of Radiation Therapy and DLN. Mice with human GBM orthotopic xenografts were treated with either mock-treatment ("Control"), DLN incorporating paclitaxel ("DLN"), radiation therapy alone ("RT") or with radiation plus DLN incorporating paclitaxel ("RT + DLN"). The survival of the mice in each treatment group is shown. The number of days following treatment associated with >50% survival of the RT only and RT + DLN groups are indicated by the grey drop-down arrows. The difference in survival between the four groups was highly significant (p<0.001).

Supplemental Tables

1.

Formulations	IC_{50} ± Standard Error [µg/ml]		
	U251 cells	SF 767 cells	T98 G cells
DLN (Tax-Filomicelles)	0.065 ± 0.005	0.063 ± 0.004	0.079 ± 0.004
Free Tax	0.064 ± 0.005	0.070 ± 0.011	0.340 ± 0.250

2.

	Days after Start of Treatment		
	0	8	17
DLN (Tax-Filomicelles)	25.1 g ± 0.56 g	25.75 ± 0.65 g	24.62 ± 0.72 g
Control	26.53 ± 1.06 g	26.66 ± 1.26 g	25.64 ± 1.02 g

3.

	Days after Start of Treatment			
	0	8	16	24
RT + DLN (Tax-Filomicelles)	23.42 ± 0.38 g	20.87 ± 0.45 g	21.93 ± 0.72 g	22.97 ± 0.74 g
RT	23.65 ± 0.50 g	20.63 ± 0.95 g	21.62 ± 0.72 g	20.79 ± 0.84 g
